# Natural language processing for aviation safety: extracting knowledge from publicly-available loss of separation reports

**DOI:** 10.12688/openreseurope.14040.1

**Published:** 2021-09-23

**Authors:** Irene Buselli, Luca Oneto, Carlo Dambra, Christian Verdonk Gallego, Miguel García Martínez, Anthony Smoker, Nnenna Ike, Tamara Pejovic, Patricia Ruiz Martino

**Affiliations:** 1 ZenaByte, Genova, Italy; 2 CRIDA, Madrid, Spain; 3 Lund University, Ljungbyhed, Sweden; 4 EUROCONTROL, Brussels, Belgium; 5 ENAIRE, Madrid, Spain

**Keywords:** ATM, Safety, Resilience, Natural Language Processing, Losses of Separation, Safety Reports, TOKAI

## Abstract

Background: The air traffic management (ATM) system has historically coped with a global increase in traffic demand ultimately leading to increased operational complexity.

When dealing with the impact of this increasing complexity on system safety it is crucial to automatically analyse the loss of separation (LoS) using tools able to extract meaningful and actionable information from safety reports.

Current research in this field mainly exploits natural language processing (NLP) to categorise the reports, with the limitations that the considered categories need to be manually annotated by experts and that general taxonomies are seldom exploited.

Methods: To address the current gaps, authors propose to perform exploratory data analysis on safety reports combining state-of-the-art techniques like topic modelling and clustering and then to develop an algorithm able to extract the Toolkit for ATM Occurrence Investigation (TOKAI) taxonomy factors from the free-text safety reports based on syntactic analysis.

TOKAI is a general taxonomy developed by EUROCONTROL and intended to become a standard and harmonised approach to future investigations.

Results: Leveraging on the LoS events reported in the public databases of the Comisión de Estudio y Análisis de Notificaciones de Incidentes de Tránsito Aéreo and the United Kingdom Airprox Board, authors show how their proposal is able to automatically extract meaningful and actionable information from safety reports and to classify them according to the TOKAI taxonomy.

The quality of the approach is also indirectly validated by checking the connection between the identified factors and the main contributor of the incidents.

Conclusions: Authors' results are a promising first step toward the full automation of a general analysis of LoS reports supported by results on real world data coming from two different sources.

In the future, authors' proposal could be extended to other taxonomies or tailored to identify factors to be included in the safety taxonomies.

## 1 Plain language summary

Nowadays, the need for automation and digitisation in the field of aviation safety is becoming crucial. In particular, this work focuses on the automated analysis of safety reports (i.e., reports describing incidents or other safety events) through different natural language processing techniques. The application of these techniques on a series of Spanish and UK reports enabled the identification of the main common topics (e.g., excessive workload), the automatic grouping of similar incidents (e.g., all the incidents originated from pilots’ unfulfillment of procedures and regulations), and the extraction of the most recurrent factors (e.g., the factor representing perception problems) according to a standard taxonomy (i.e., the Toolkit for ATM Occurrence Investigation).

## 2 Introduction

The air traffic management (ATM) system has historically coped with a globally increasing traffic demand, and this growing amount of flights has ultimately led to increased operational complexity ^1^. According to the Single European Sky (SES) European ATM Masterplan ^2^, the increased complexity of the ATM system should be absorbed by increased deployment of automation solutions in order to achieve a more efficient and safe traffic management.

On one hand, the growth in demand is accompanied by an increase in the size of data that needs to be taken into account in the safety management operations and, on the other hand, today’s safety events are gradually becoming of a more complex and uncommon nature than those of yesteryear ^3^. As such, attempts to understand and prevent these safety events require more detailed knowledge of underlying dynamics.

The FARO project — saFety And Resilience guidelines for aviatiOn — focuses on the problem of dealing with the impact that an increasingly complex environment has on the system safety. In this work, which is framed within the context of the FARO project, the focal research subject is a specific manifestation of ATM safety, the loss of separation (LoS), and in particular the analysis of the LoS safety reports produced by states’ Civil Aviation Authorities and Air Navigation Service Providers (ANSPs) after investigation.

The reports considered in this study are specific to Spanish and UK airspaces, and are collected in the public databases of, respectively, the Comisión de Estudio y Análisis de Notificaciones de Incidentes de Tránsito Aéreo ( CEANITA). and the UK Airprox Board ( UKAB).

In general, safety reports are extremely valuable sources of data to learn from past incidents, as well as to identify new threats to safety and ways to avoid them ^4^. However, manual analysis of these reports is complex and requires considerable resources. Each safety report is composed mainly of free text in natural language, which makes it difficult for automated tools to process them. This work exploits natural language processing (NLP) towards automation in the analysis of safety reports.

In the last two decades, the application of NLP to safety reports has been increasingly explored, but research has mainly focused on developing models and algorithms to categorise incident reports ^5– 8^. All of these works rely on an initial set of labels and training data consisting of safety reports previously labelled by domain experts. The biggest limitation of this approach is its lack of generality: to generate a new set of labels and training data, substantial resources and effort would be needed. In this context, the importance of introducing common sets of labels — i.e., a common taxonomies — became evident. On one hand, tools like the Toolkit for ATM Occurrence Investigation (TOKAI) have been developed to generate structured safety data ^9^, and the data collected have proved to be extremely useful for quantitative analysis ^10^; on the other hand, some applications of NLP techniques have focused on the categorisation of the safety reports according to taxonomy factors ^11^. However, the categorisation approach shows another limitation: while it is a good way to automatise a task performed by domain experts, this approach does not allow the discovery of unknown patterns or further knowledge. To overcome this limitation, unsupervised techniques like topic modelling ^4, 12– 14^ and similarity clustering ^4, 15^ are now being explored.

To address current gaps in the literature, in this work the authors propose a twofold approach:

- An unsupervised phase: an exploratory data analysis (EDA) is performed on safety reports combining state-of-the-art techniques like topic modelling and clustering;
- A partially supervised phase: for the first time, an algorithm able to extract TOKAI taxonomy factors from the free-text safety reports is developed, based on syntactic analysis.

Both these phases focus on the mining of free text contained in LoS reports in order to identify — via topic modelling and clustering — and categorise — via the TOKAI-taxonomy-extraction algorithm — common patterns of behaviour. In particular, topic modelling and clustering act in an unsupervised fashion, enabling the detection of possibly unknown recurrent behaviours or conditions during LoS events, while the application of syntactic analysis allows the association of predetermined patterns of behaviour to TOKAI taxonomy factors (e.g., perception, conformance to procedures, or memory). An analysis of the importance of combining unsupervised approaches and taxonomy exploitation as well as the main limitation of both methodologies can be found in 16. The choice of the TOKAI taxonomy for the second phase is based on three main reasons.

First, the TOKAI taxonomy makes a significant shift from traditional causal taxonomies based on negative perspectives (i.e., describing errors or failures) by its use of neutralised language: TOKAI factors are neither negatively nor positively oriented, so they can ideally explain both ordinary operational situations and safety occurrences ^10^. As a consequence, this allows the framing of the identified behaviours in a Resilience Engineering (RE) perspective: successes and failures do not emerge from different pathways in the work system, but the same set of conditions can evolve to either ^17, 18^. The second reason is of a more practical nature: the structure of TOKAI taxonomy is particularly suited to allow aggregation at different levels of detail, given its multi-level hierarchical structure. Lastly, the TOKAI taxonomy is intended to become a standard and harmonised approach to future investigations, allowing ANSPs to share lessons from ATM occurrences ^10^. As such, extracting the same factors from past reports may be useful to align the past analyses with the future ones.

The rest of the paper is organised as follows. Section 3 details the scope of this work. Section 4 describes the available data used to test the methodologies (presented in Section 5). Section 6 reports on the results from the application of the proposed methodologies on the available data. Finally, Section 7 concludes the paper.

## 3 Scope of the work

The scope of this work is to facilitate the extraction of meaningful and actionable information from recent (i.e., between 2017 and 2019) CEANITA and UKAB LoS reports, and, in particular, to automatically identify recurrent behaviours and common precursors. More specifically, a twofold approach was applied:

- First, an EDA was performed in order to get general insights into the LoS phenomena (see Section 3.1);
- Then, an algorithm able to extract selected TOKAI taxonomy factors from the free text of CEANITA reports was developed, based on syntactic analysis (see Section 3.2).

### 3.1 Exploratory data analysis

The exploratory data analysis was conducted in two stages.

In the first stage, the most recurrent topics in the corpus of both CEANITA and UKAB LoS reports were identified exploiting topic modelling ^19^. Topic modelling is an unsupervised NLP technique able to automatise the extraction of the most recurrent topics and compute their prevalence in each report. This technique enables a high-level analysis of the content of each report, which can therefore be described through numerical features and possibly compared in a scalable way, without the need to read and understand them one by one.

In the second stage, a cluster analysis ^20^ is applied to group similar LoS events in terms of the various themes or safety areas contained in the reports (i.e., topics prevalence, main causes of the incident, safety barriers, etc.).

### 3.2 Automatic extraction of TOKAI taxonomy factors

Each CEANITA report concludes with a free-text description of the actions performed by air traffic controllers (ATCos) and pilots that contributed to the incident. Analogously, in a sample of UKAB reports, the final assessment of cause is listed in free text and includes the main contributory factors based on pilots’ and controllers’ actions. This information is crucial to understanding the dynamics at play in the LoS. Therefore, the automatic extraction and classification of these contributing behaviours can be of paramount importance. In particular, labelling these behaviours according to a standard taxonomy — in our case the TOKAI one — can enable the application of quantitative-analysis techniques ^10^ on information extracted from various reports and/or repositories.

Syntactic analysis is a branch of NLP which focuses on determining the grammatical structure of a sentence. State-of-the-art tools for syntactic analysis ^21^ are able to identify base-form verbs (e.g., the group of verbs related to TOKAI factor A.1: “detect”, “identify”, “see’, “hear”, etc.), as well as to retrieve information about their role in the sentence: their form (e.g., active or passive) and their subject. Thus, each action can be potentially associated to a TOKAI taxonomy factor, whilst maintaining information about who performed the action.

After extracting these taxonomy factors it is possible to estimate the occurrences of each factor in the corpus of reports. Consequently, relying on this information, a sort of sanity check can be performed to validate this syntactic-analysis approach: a simple model is developed to predict whether the main responsibility of the incident is ascribed to the ATCo, pilot, or both. In fact, the more reliable the extracted information is, the more reasonable it is to assume it should be predictive of the main contributor(s) of the incident. Whilst this is not a direct validation of the model, the outputs are indicative of the reliability of the algorithm.

## 4 Data description

For the scope of this study, two publicly-available data sources were exploited: CEANITA reports (see Section 4.1) and UKAB reports (see Section 4.2).

### 4.1 CEANITA LoS reports

The considered CEANITA LoS reports consist of 89 safety reports, written in Spanish and published by Spanish Safety Aviation Agency (AESA), which is the Spanish Civil Aviation Authority, under the commission of CEANITA, covering safety-related occurrences in the Spanish airspace between January 2018 and July 2019.

The initial sections of these reports are written in fixed formulas or tabular format. This enables the direct extraction of some categorical or numerical variables through an automated search for keywords or table margins, and therefore the computation of some basic descriptive statistics such as:

- the *ICAO risk category*: 9% of the occurrences are classified as A, while the majority is classified as B (55.1%) and C (31.5%), and only 3.4% as D and 1.1% as E (classes assigned according to the ICAO classification ^22, 23^);
- the *main causes*: the most frequent ones in the corpus are wrong clearance (52%), deviation from procedures (22%), wrong or no resolution (17%–15%), coordination problems (17%), and late or no detection (15%–16%) — note that multiple causes are possible;
- the *airspace class*: most of the reported incidents happened in class C, D (40% each), and A (11%), while only 6% in G and 3% in E (classes assigned according to the ICAO classification ^24^);
- the *pilots and ATCo contribution*: pilots’ contribution is classified as direct in 36% of the cases, as indirect in 15%, and as none in 49%. ATCo contribution is, instead, direct in the majority of cases (72%), indirect in 9% of the incidents, and none in 19%.

The remaining part of each report is written as a free text and divided in the following sections:

- *Initial situation*: in this section the initial situation (i.e., the initial location and condition of the aircraft involved in the LoS) is described with text and images.
- *Communications and radar tracks*: in this section the communications of interest between ATCos and pilots are summarised.
- *Extract from received reports*: this section is a summary of the different reports received by the commission of investigation (i.e., from the pilot, the co-pilot, the executive controller, and other involved agents, if any).
- *Conclusions*: this section describes the conclusions of the investigation, summarising the dynamic of the LoS based on the main actions performed by the involved human actors.

### 4.2 UKAB LoS reports

The considered UKAB LoS reports consist of 549 safety reports, written in English and published by UKAB, covering safety-related occurrences in the UK airspace between January 2017 and December 2019.

**Figure 1.**
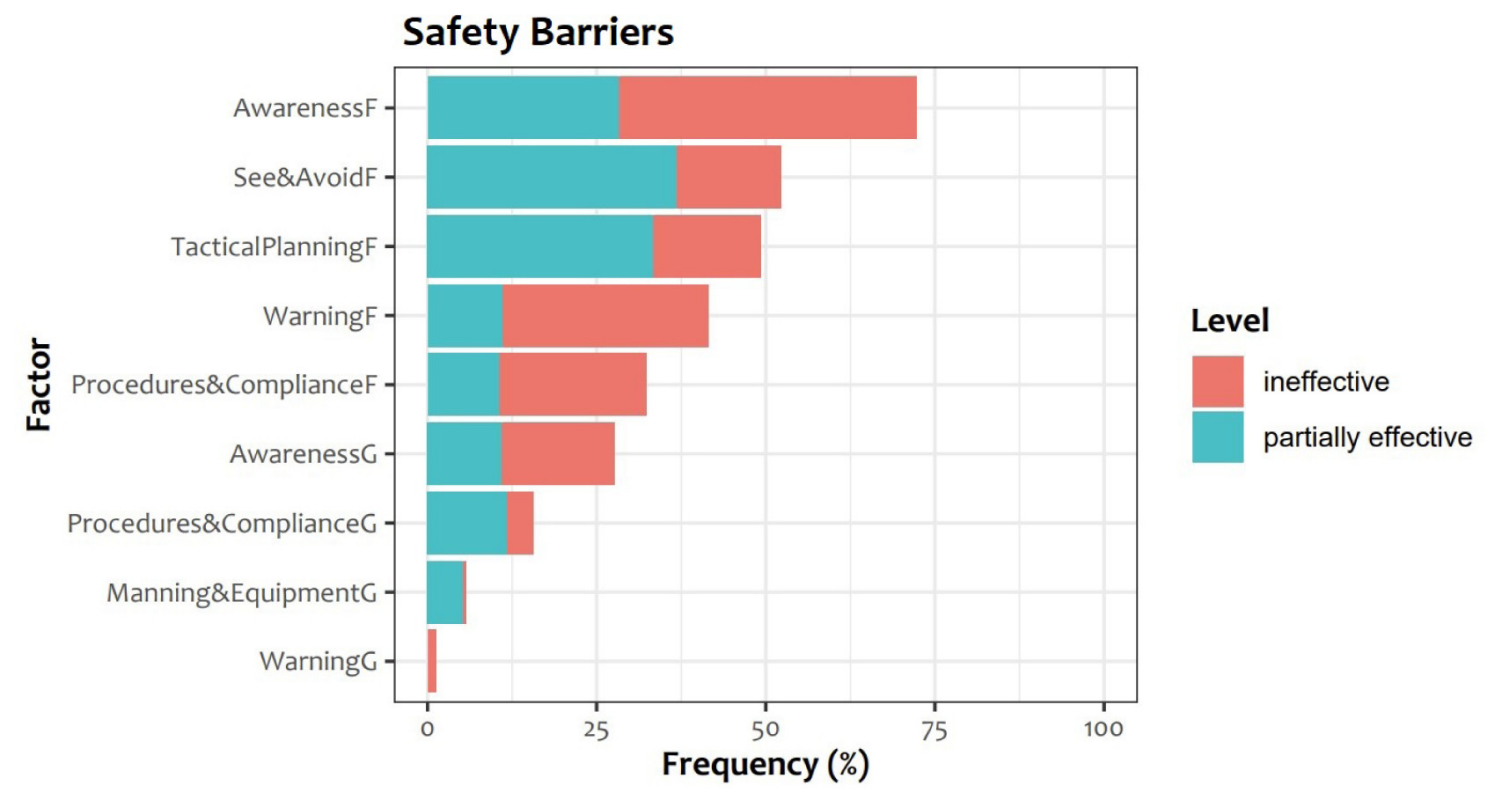
Frequency of ineffectiveness and partial ineffectiveness of Safety Barriers in UKAB reports.

Similarly to the CEANITA reports, some sections of these UKAB reports are written in fixed formulas or tabular format, so that some information can be directly extracted, such as:

- the *ICAO risk category*: 9.3% of the events are classified as A, 26.8% as B, 48.5% as C and 2.2% as D, while 13.3% are classified as E, which means a non-negligible part of the reports describe situations that were not actual incidents;
- the *airspace class*: most of the reported incidents happened in class G (89.1%), while 7.3% in class D, 2.2% in class A and 1.3% in class C;
- the *safety barriers*: their distribution in the considered reports can be seen in Figure 1 and a detailed explanation of each single barrier is available from AIRPROX.

The remaining part of each report is written as a free text and divided in the following sections:

- *Information reported to UKAB*: this section summarises the different reports received by the Airprox Board, not only from the pilot, the executive controller, and the other directly involved agents, but also from UKAB Secretariat and experts commenting on the event;
- *Board’s discussion*: this section describes the Board’s discussion and its conclusions, motivating each consideration based on the information available;
- *Assessment of cause and risk*: this section briefly summarises the main causes, contributory factors and the effectiveness of Safety Barriers as identified by the Board. Safety Barriers are described in Table 1 and the distribution of their effectiveness is shown in Figure 1.

**Table 1.** Legend of the safety barriers considered in UKAB reports for cause assessment, together with the abbreviations used in Figure 1.

Barrier	Abbreviation
GROUND ELEMENTS	
Regulations, Processes, Procedures and Compliance	Procedures&ComplianceG
Manning & Equipment	Manning&EquipmentG
Situational Awareness of the Confliction & Action	AwarenessG
Electronic Warning System Operation and Compliance	WarningG
FLIGHT ELEMENTS	
Regulations, Processes, Procedures and Compliance	Procedures&ComplianceF
Tactical Planning and Execution	TacticalPlanningF
Situational Awareness of the Conflicting Aircraft & Action	AwarenessF
Electronic Warning System Operation and Compliance	WarningF
See & Avoid	See&AvoidF

## 5 Methods

This section presents the theoretical framework of the methods exploited to achieve the scope of the work (see Section 3) leveraging the data described in Section 4. Four main techniques were exploited: topic modelling ( Section 5.1), clustering analysis ( Section 5.2), syntactic analysis ( Section 5.3), and data-driven predictive models ( Section 5.4).

### 5.1 Topic modelling

Topic modelling is an unsupervised NLP technique designed for the first time by David Blei and John Lafferty ^19^, and largely used in the transportation domain ^25, 26^. The idea behind topic modelling is to represent a corpus of documents in terms of a certain number of topics, identified in a completely unsupervised fashion, based only on how the words are distributed in the documents. For these characteristics, this method is particularly suited to outline the main themes in a collection of documents. The statistical intuition behind topic modelling can be summarised in three points:

- A document can be defined as a set of words.
- A document contains different topics according to a certain distribution.
- A topic can be in turn defined through a certain distribution of words prevalence.

Thus, by observing the frequencies of words in a collection of documents, it is possible to estimate the two underlying distributions fitting the observed frequencies. In particular, the most widely used technique for topic modelling, the latent Dirichlet allocation (LDA), is characterised by the theoretical assumption that these distributions can be derived based on the Dirichlet probability distribution ^4, 12^. This framework also generates a topic-word matrix in which each topic is represented through weights associated to each word. This information can be used to interpret the (otherwise unlabelled) topics. In this work, for both the use cases, the LDA models were developed using the textmineR library version 3.0.5 from R development environment version 4.0.3. The number of iterations for the Gibbs sampler to run was set to 500 and the burn in was set to 180, while every 10 iterations of the sampler alpha was set to be optimised and the likelihood to be re-computed. The other number *k* of topics to be generated was tuned by testing different options and evaluating the results in terms of probabilistic coherence and R-squared (in particular, for the CEANITA use case *k* = 27 and for the UKAB one *k* = 60). The final number of meaningful and significant topics to be considered was manually identified to be 12 for the CEANITA reports and 23 for the UKAB reports, according to the experts of the field involved in this work.

### 5.2 Clustering analysis

Clustering analysis ^20^ is a technique used to group data according to a certain definition of similarity. In particular, hierarchical clustering is one of the most largely exploited methods ^27^. In this context, the agglomerative hierarchical clustering, as opposed to the divisive one, has been shown to be the most effective ^27^, and is therefore the one used in this work. The idea behind the agglomerative hierarchical clustering is the following. Initially, each point in the dataset is separate from the other and considered as an individual cluster. Then, each cluster is merged with other clusters based on their mutual distance, where the definition of distance is chosen according to how well it describes the concept of (dis)similarity in the considered data (e.g., correlation-based distances are often used in gene expression data analysis). Keeping track of each step of the process, the clusters are thus merged until all the data converge to a single cluster. Finally, the user selects the best number of clusters based on the knowledge of the subject, or the intra-cluster variability, or other particular statistical metrics ^28^.

In this case, data were merged according to Ward’s minimum variance criterion (i.e., the distance between objects is proportional to the squared Euclidean distance, which is the standard for most applications), using the basic stats library version 0.1.0 from R development environment version 4.0.3, after normalising all the numerical variables (and after mapping the qualitative variables of interest into numerical features, i.e., the main cause and the contribution for the CEANITA reports and the safety barriers for the UKAB ones). The choice of the number of clusters was made considering both experts’ knowledge of the field and statistical metrics, in particular the dendrogram and the scree plot.

### 5.3 Syntactic analysis

Syntactic analysis deals with the problem of analysing a string in natural language to identify the syntactic and grammatical structure of each sentence. In this work, syntactic analysis is performed using the UDPipe library version 0.8.6 from R development environment version 4.0.3, a state-of-the-art open-source library which automatically generates sentence segmentation, tokenisation, part-of-speech tagging, lemmatisation, and dependency parsing. Models are provided for 50 languages. An example of the output of the UDPipe library can be found in Table 2. A detailed explanation of the tool can be found in 21.

**Table 2.** Example of syntactic analysis with UDPipe for the sentence “The radar controllers did not issue timely traffic information”. The meaning of “Part of Speech” and “Dependency” elements is standard ^a^.

Sentence	Lemma	Part of Speech	Dependency
The	the	det	det
radar	radar	noun	compound
controllers	controller	noun	nsubj
did	do	aux	aux
not	not	part	advmod
issue	issue	verb	root
timely	timely	adj	amod
traffic	traffic	noun	compound
information	information	noun	obj

^a^ https://universaldependencies.org/u/dep/all.html

### 5.4 Data-driven models (for validation of Algorithm 1)

Data-driven predictive models are based on the idea of learning relations between inputs (e.g., taxonomy factors prevalence extracted by Algorithm 1) and outputs (e.g., LoS direct contribution reported in the reports) through a series of examples (i.e., historical data). This will serve as validation of the quality of the taxonomy factors prevalence extracted by the proposed Algorithm 1.

In this context, support vector machines (SVMs) ^29^ represent state-of-the-art solutions for many real-world applications ^30, 31^ in the framework of (shallow) machine learning algorithms. Even if, currently, deep learning approaches ^32^ were shown to outperform shallow learning models in many tasks (e.g., vision and speech recognition), they require very large amounts of data to be trained, which were not available for this research.

SVMs are the most effective algorithms in the family of Kernel Methods ^29^ (i.e., methods exploiting the “kernel trick” to extend linear techniques to the solution of nonlinear problems). SVMs have a series of hyperparameters—the kernel, which is often fixed to be the Gaussian one ^33^, the kernel hyperparameter, and the complexity hyperparameter — which deeply influence their performance and need to be tuned during the model-selection phase ^34^.

However, data-driven predictive models need not only to be tuned (by finding the optimal hyperparameters) but also to be evaluated in terms of their performance in a rigorous statistical way. Model selection and error estimation are meant to deal exactly with this problem ^34^. Resampling techniques like k-fold cross validation and non-parametric bootstrap are between the most commonly exploited solutions, since they are proved to work well in many situations ^34^. The idea behind these techniques is simple: the original dataset is re-sampled once or more, without replacement, to build three independent datasets called learning, validation, and test set. The learning set is exploited to train the model. The validation set is exploited to find the optimal hyperparameters (i.e., the ones that lead to the optimal performance). The test set is exploited to estimate the performance of the final model: in this way, the test is independent from both the learning and the validation.

Performance measures strongly depend on the task to be solved. In this case, dealing with classification problems (see Section 6), accuracy and confusion matrix are the most widely used metrics ^35^.

## 6 Results

This section shows how the methods presented in Section 5 have been applied to achieve the scope of the work (see Section 3) demonstrating the effectiveness of the proposed approach on both the sets of data described in Section 4. Specifically, Section 6.1 presents the results of EDA, summarising the main outcomes produced by topic modelling and clustering analysis, while Section 6.2 presents the results of the syntactic analysis approach used to connect the reports with the TOKAI taxonomy, also validating the quality of the methodology.

### 6.1 Exploratory data analysis

This section shows how topic modelling (see Section 5.1) can be used to extract the main topics from the 89 CEANITA reports and the 549 UKAB reports and how clustering analysis is able to group the LoS events in a meaningful way, in both the sets of data considered.

***6.1.1 Topic modelling on CEANITA reports***. The application of LDA for topic modelling on CEANITA reports led to the identification of 12 main topics. These 12 topics can be defined by lists of words and bigrams, to which the FARO experts have associated representative labels (see Table 3). The selection of the topics was performed through both automated procedures (i.e., relying on coherence metrics) and more handcrafted adjustments (i.e., consulting the FARO experts, which filtered the topics and retained the most meaningful and coherent ones according to their knowledge of the domain).

**Table 3.** Words and bigrams of the 12 topics extracted with LDA from CEANITA reports, together with the representative label associated to each topic by FARO’s experts (English translation from Spanish).

Words/Bigrams							Topic
helicopter	drop	water	fires	extinguishing	coordination	drop area	fire
load	work	high	alone	workload	instructions	previous	workload
departure	to take off	aircraft climb	runway	to take off aircraft	rate	they are	departure
wind	tail	down-wind	leg	wind leg	right tail	runway	traffic circuit
weather	adverse	adverse weather	detours	meteorologic conditions	due to weather	thunderstorm	weather
runway	go around	go	around	to take off	to land	aircraft established	go around
sectors	sector aircraft	frequency sector	high	coordination	transfer	limit	sector operations
answer	received	finally	decided	they saw	communication	visual contact	communication
clearance	course descent	aircraft to descend	descent rate	sector to descend	aircraft to maintain	rate	descent
received	coordinating	confirming	to confirm receipt	maintaining formation	sector informs	receipt	coordination
alert	early	early alert	activation function	activation	function	alert function	alert
military	military formation	formation	military aircraft	defence	air defence	main centre	military

**Figure 2.**
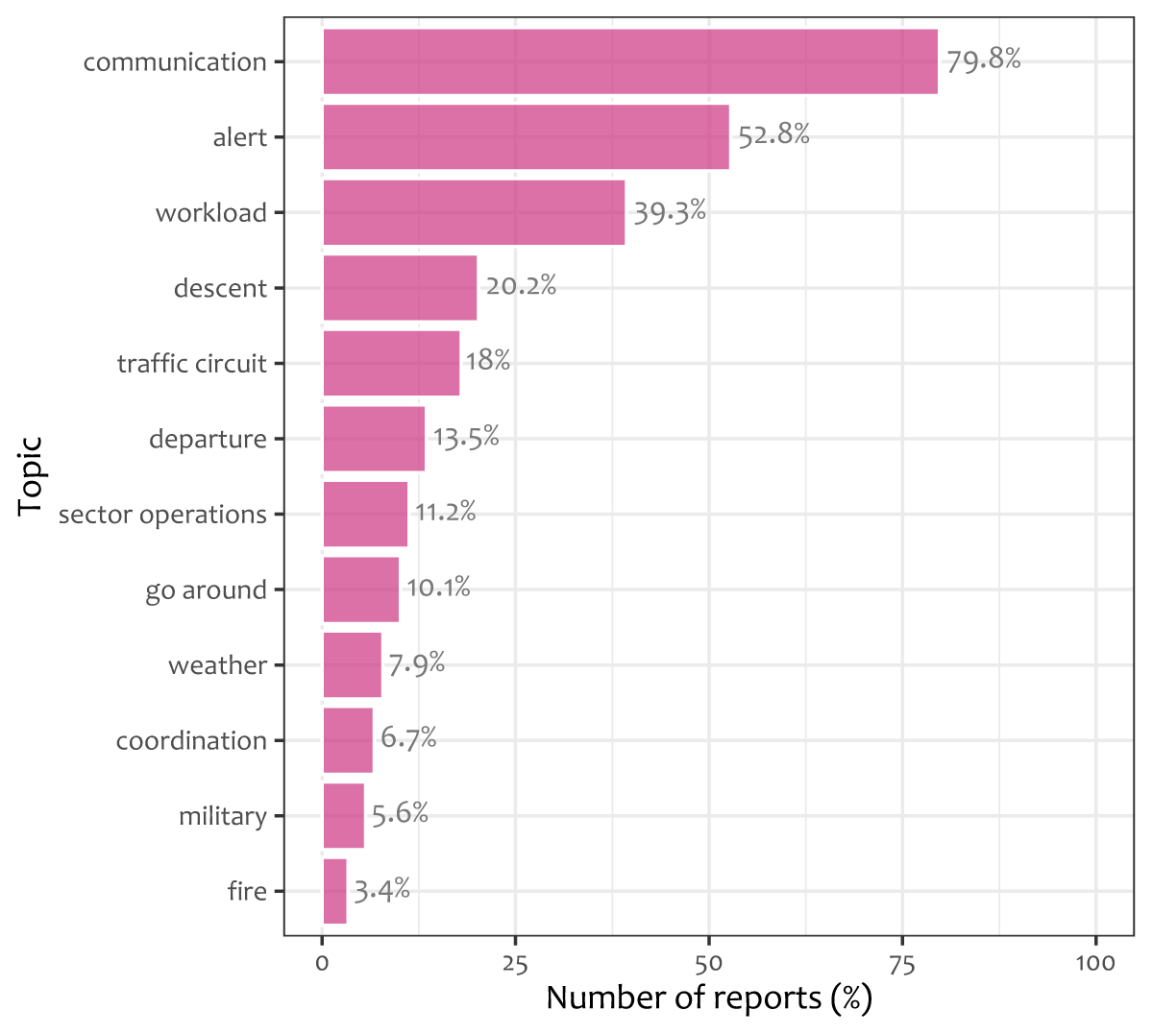
Average prevalence of each of the 12 topics of Table 3 over CEANITA reports.

Topic modelling results enable the description of the reports at a higher resolution then simple descriptive statistics. For instance, while the main causes of the incident are identified just by looking at variables described in Section 4.1 (e.g., if a wrong ATCo clearance was responsible), topic modelling also provides additional information (e.g., if the ATCo’s behaviour that led to the wrong clearance might have been affected by an excessive workload or an emergency situation). Indeed, this technique also outputs the probability of finding a certain topic—namely, the topic’s prevalence — in each report, thus generating for every document a set of numerical features quantitatively describing its content. Figure 2 shows the average prevalence of each topic over the 89 CEANITA reports. Observing Figure 2 it can be seen that exogenous factors like fire-extinguishing emergencies or adverse-weather problems are quite rare (only about 10% of the incidents contain one of these topics), while workload is present in almost 40% of the reports.

**Figure 3.**
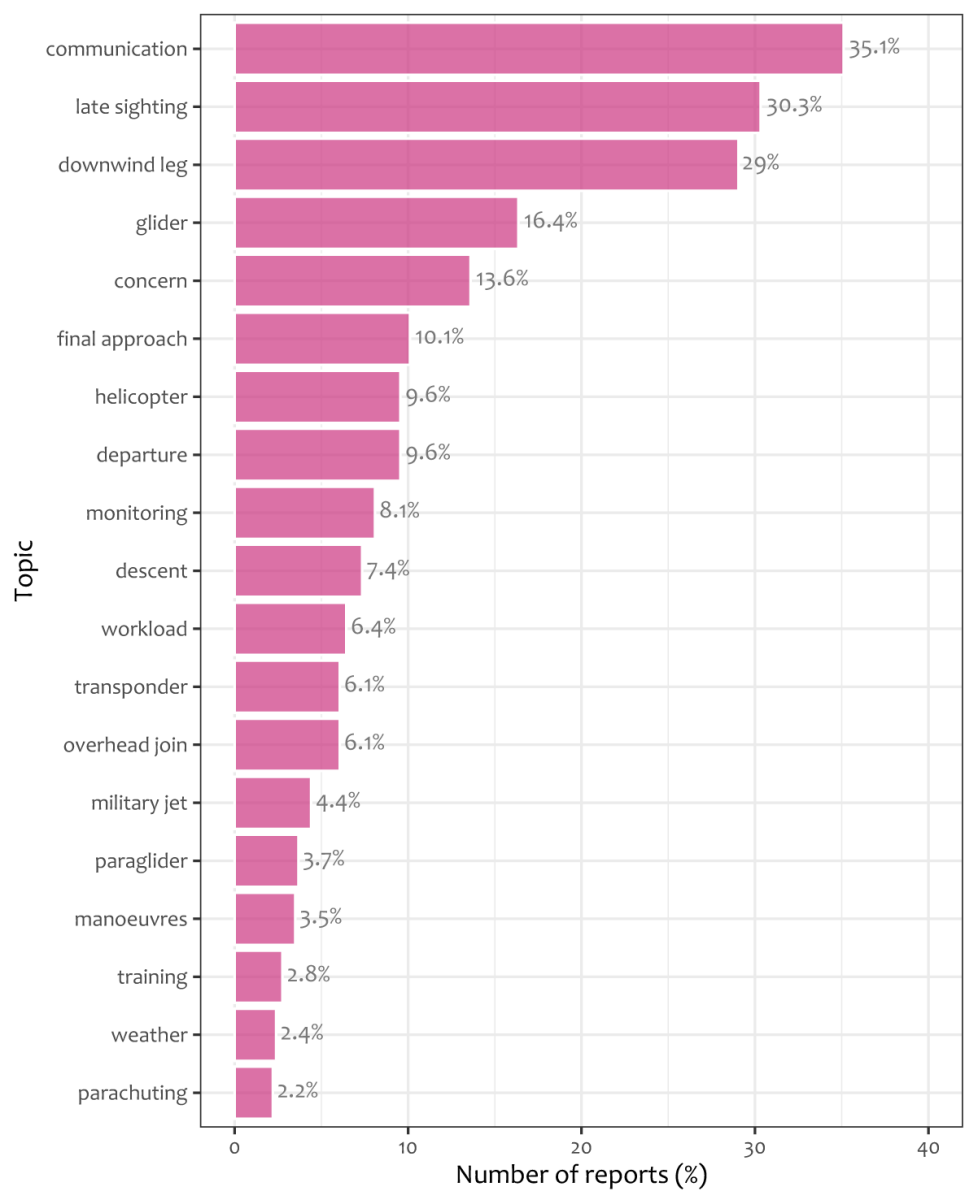
Average prevalence of each of the 20 topics of Table 4 over UKAB reports.

***6.1.2 Topic modelling on UKAB reports***. The application of LDA for topic modelling on UKAB reports led to the identification of 20 main topics, whose description in terms of words and bigrams in Table 4. Figure 3 shows the average prevalence of each topic over the 549 UKAB reports. There is some significant heterogeneity in prevalence: for instance, communication, late sighting, and downwind leg are quite frequent (around 30%) while topics about parachuting, weather or training are quite rare (around 2%).

**Table 4.** Words and bigrams of the 20 topics extracted with LDA from UKAB reports, together with the representative label associated to each topic by FARO’s experts.

Words/Bigrams							Topic
overhead	join	joining	deadside	overhead join	crosswind	circuit	overhead join
student	instructor	student pilot	training	solo	hand	control	training
advised	requested	acknowledged	asked	received	inbound	passed pilot	communication
fast	military	jet	high	fast jet	range	manoeuvres	military jet
survey	manoeuvring	company	operations	conducting	aerobatics	manoeuvres	manoeuvres
runway	go around	go	around	to take off	to land	aircraft established	go around
sectors	sector aircraft	frequency sector	high	coordination	transfer	limit	sector operations
answer	received	finally	decided	they saw	communication	visual contact	communication
site	gliding	winch	glider	launch	active	sites	glider
departure	departing	climb	climbing	airborne	departed	depart	departure
converging	sighting pilot	required give	late sighting	pilot required	considered converging	converging pilot	late sighting
descent	descend	descending	altitude	descended	feet	vertical	descent
trainee	ojti	handover	clearance	instruction	cleared	training	training
helicopter	site	helicopters	helicopter pilot	wing	landing	lifting	helicopter
service	altitude	cloud	weather	receiving	altitude ft	condition	weather
transponder	primary	primary contact	twin	selected	serviceable	equipped	transponder
approach	final	runway	instructed	landing	final approach	leg	final approach
monitor	traffic information	required monitor	warning	definite risk	monitor flight	definite	monitoring
para	drop	parachuting	dropping	parachute	site	para dropping	parachuting
busy	workload	high	working	handover	controlling	inbound	workload
paraglider	paramotor	flypast	paragliders	paraglider pilot	paramotor pilot	paraglider pilots	paraglider
concerned	normal	concerned proximity	standards	pilot concerned	safety standards	pertained	concern
circuit	downwind	visual circuit	leg	pattern	ahead	circuit traffic	downwind leg

**Figure 4.**
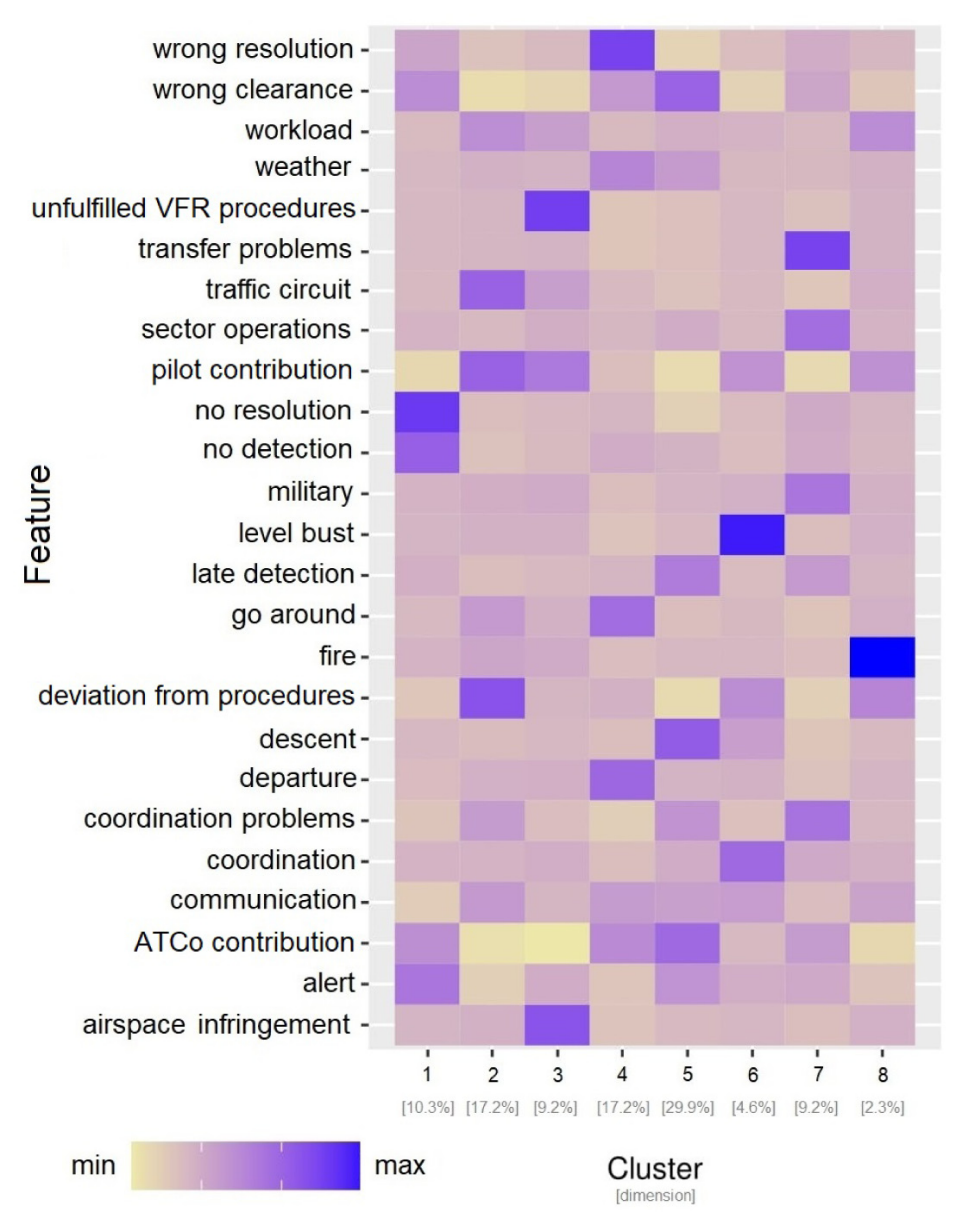
Characterisation of the 8 clusters in CEANITA reports through the size and the normalised relative frequency of each feature.

***6.1.3 Clustering analysis on CEANITA reports***. In order to identify the relations between the topics’ prevalence and the other contextual information (i.e., the main causes of the LoS and the pilots’ and ATCo’s contribution to the incident), a further analysis was then conducted by applying clustering analysis (see Section 5.2). For this purpose, for each CEANITA report, a feature set was created, composed of the prevalence of the 12 topics, the main causes, and the level of pilots’ and ATCo’s contribution to the LoS. Hierarchical clustering with Ward distance was then applied on the resulting dataset. After visualising different statistical metrics through dendrograms and screeplots (i.e., the two most common methods for cluster selection) and jointly consulting the FARO experts, 8 different clusters were identified. As shown in Figure 4, they strongly differ in size and normalised frequency of the features. Indeed, looking at Figure 4 some observations arise:

- Two very small subgroups (Clusters 6 and 8) are identified as particularly different from the others. In particular, Cluster 8 is composed of two LoS events where the main topic is “fire” (indeed, they are the reports referred to Llutxent fire in summer 2018); Cluster 6 instead contains the four incidents caused by level bust (and, as expected, according to the heatmap, the main contribution was from the pilots and the other main conclusion was “deviation from procedures”).
- The largest cluster (Cluster 5) is mainly composed of wrong-clearance and late-detection incidents, with clearly the highest frequency of ATCo contribution and an interesting high prevalence of “descent” topic.
- Cluster 4 contains incidents mainly caused by “wrong resolution” of the ATCo, with high prevalence of topics related to go-around, departure, and weather.
- Cluster 7 is composed of incidents caused mainly by transfer or coordination problems. The most frequent topics here are “sector operations” and “military”.
- Incidents in Cluster 3 are essentially due to Pilots’ errors, in particular to airspace infringement and unfulfillment of the Visual Flight Rules (VFR).
- Cluster 2 is characterised by incidents due to Pilots’ deviations from procedures, especially in the landing phase (see topic “traffic circuit”).
- Cluster 1 is composed of incidents due to ATCo inability to both detect and resolve the LoS. This cluster is interestingly characterised by high values of the topic “alert”;
- Interestingly some topics (e.g., workload and communication) are almost homogeneously distributed in all the clusters, without peaks in their relative-frequency values.

**Figure 5.**
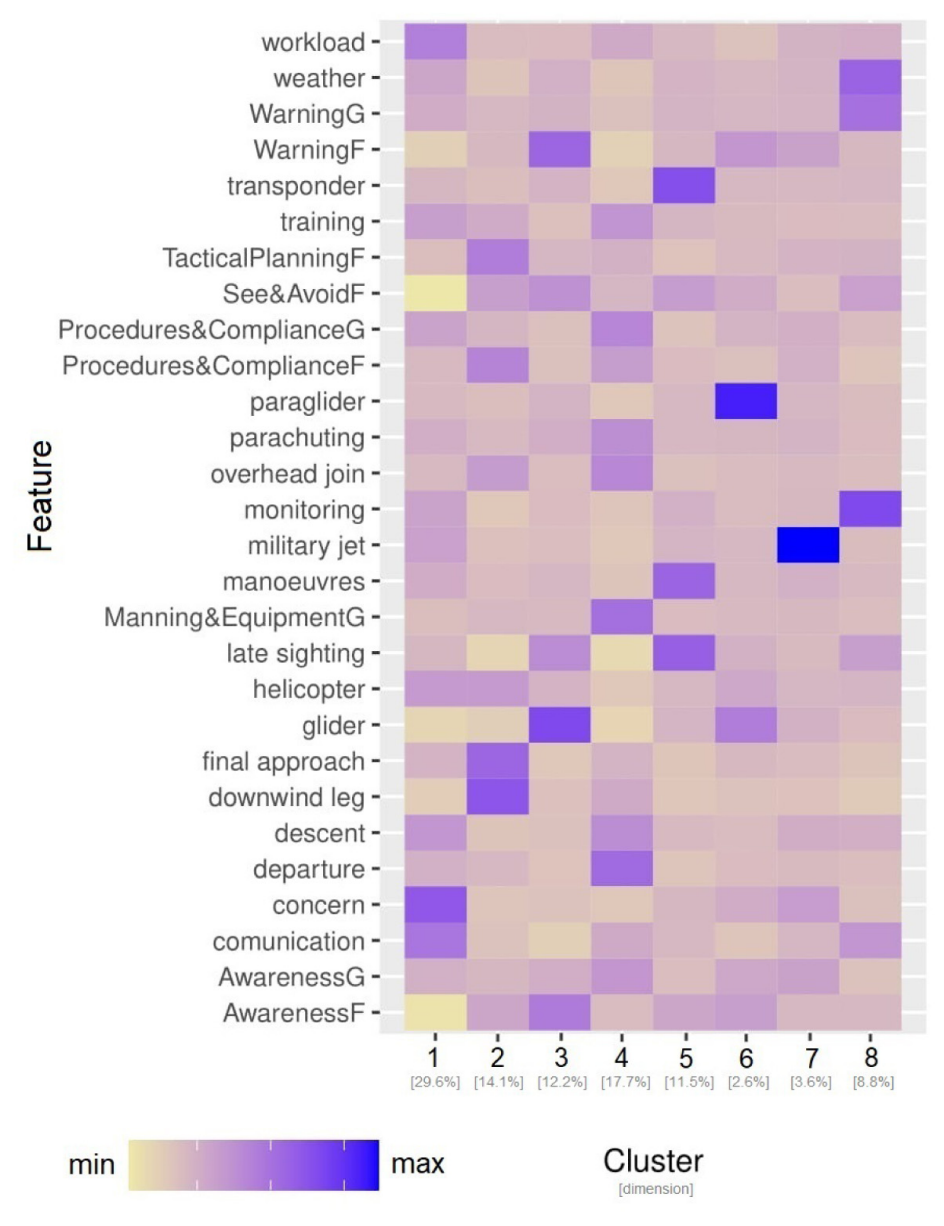
Characterisation of the 8 clusters in UKAB reports through the size and the normalised relative frequency of each feature.

***6.1.4 Clustering analysis on UKAB reports***. An analogous analysis was conducted on UKAB reports: the feature set in this use case is composed of the prevalence of the 20 topics and the safety barriers. Also in this corpus 8 clusters were identified, differing in size and features (see Figure 5). Figure 5 shows that:

- Also in this use case, two relatively small clusters (Cluster 6 and Cluster 7) are easily identifiable as sort of “outliers”: one is essentially described by the absolute prevalence of the topic “paragliders” and the other one of the topic “military jets”, without other particularly evident features.
- Cluster 8 is interestingly characterised by three main topics/factors: weather, monitoring and electronic warning system at ground level, which may be correlated in some way.
- Cluster 1, even being the largest one, seems to reunite the incidents where the responsibility is rarely attributed to the pilot: most of the Safety Barriers related to flight level have lower levels than the average, while the darkest colours on the heatmap correspond to concern, communication, and workload, highlighting a significant human component.
- Cluster 2 is mainly formed by incidents happened during landings, with a slight correlation with procedures compliance, tactical planning and execution ascribed to pilots.
- Cluster 3 mostly contains late-sighting incidents, correlated with factors like lack of situational awareness of the pilot and electronic warning problems; interestingly, this cluster is the one with higher prevalence of the glider topic.
- Cluster 4 is formed by low-altitude incidents: the heatmap highlights both departure, descent, overhead join, and parachuting topics; the main causal factor seems to be Manning & Equipment at ground level.
- Cluster 5 is again about late-sighting incidents, with particular focus on two topics: transponder and manoeuvres.

### 6.2 Automatic extraction of TOKAI taxonomy factors

Syntactic analysis (see Section 5.3) is a powerful tool to identify the text structure and meaning, and, in particular, in this work it enabled the association of each considered report to the corresponding TOKAI taxonomy factors. Specifically, for the purposes of this research, only Part A of the TOKAI taxonomy was exploited (i.e., the one related to the personnel), since the actions reported in the paragraphs of interest from both the CEANITA and the UKAB reports are usually more related to this subject. To have a clearer picture of the considered taxonomy factors, Table 5 reports Part A of the TOKAI taxonomy together with factors’ specifications ^10^ and examples of sentences associated to them through the algorithm developed by the authors.

**Table 5.** Part A of the TOKAI taxonomy factors: specifications and examples of sentences associated to the taxonomy by the developed tool.

Factor	Specifications	Example
A-1. Perception	See - identification; See - detection; Hear - identification; Hear - detection; Perceive visual information - accuracy; Perceive auditory information - accuracy.	Sector CAO authorised aircraft 1 without detecting aircraft 2.
A-2. Memory	Remember to monitor or check; Remember to act; Remember previous actions; Recall information from working memory; Recall information from long-term memory.	Aircraft 2 was authorised by the Sector, not remembering presence of Aircraft 1.
A-3. Decision	Judge/Project; Decide/Plan.	APP LEMG planned the approximation sequence incorrectly.
A-4. Action	Select/Position manually; Convey/Record information.	Aircraft 1 did not communicate its position correctly.
A-5. Conformance	Deliberate or malicious act; Individual conformance with rules or procedures; Team conformance with rules or procedures.	Aircraft 2 did not comply with the instruction.

The algorithm to link the report text to the TOKAI taxonomy factors can now be illustrated in detail (see Algorithm 1).

***6.2.1 Outcomes on CEANITA reports***. The proposed algorithm ( Algorithm 1) has then been applied to the conclusive section of each CEANITA report. This portion of text summarises the dynamics of the LoS based on the main actions of pilots and ATCos. There follows an example from report 067/18 (translated from Spanish to English):

“According to what stated by the executive controller of ACC Barcelona Sector CCC, different conflicts were going on: due to this, he did not plan the descent of Aircraft 1, which ended up in conflict with the trajectory of Aircraft 2. When Aircraft 1 asked for descent, he did not check if the traffic around Aircraft 1 was in potential conflict with it and authorised it to descend at FL310. This produced the loss of separation between Aircraft1 and Aircraft 2. The planning controller of Sector CCC immediately informed the executive controller of the conflict, but this did not prevent the airprox.
On the other hand, Sector CCC provided incomplete traffic information to Aircraft 2.”

**Algorithm 1: Algorithm to link the report text to the TOKAI taxonomy factors exploiting Syntactic Analysis**

**Input: 1.** The sequences of verbs/actions in the base form for each factor (e.g., for factor A-1, the list “see”, “identify”, “detect”, “hear”, etc.). This sequences can be created directly by human operators, which can be supported by automatic tools. Possibly, two sequences can be created for each factor, a positive and a negative one (e.g., for A-2, “remember” is in the positive sequence, while “forget” in the negative one).

            
**2.** The text of the conclusive section of the report of interest.

**Output:** For each of the factors (i.e., A-1, A-2, etc. in Table 5) and for each subject (e.g., pilot or controller) the number of positive and negative occurrences.

The text of the report is processed via UDPipe (see Section 5.3 and Table 2 as reference);

In the UDPipe output (i.e., the result of lemmatisation, part-of-speech tagging, and dependency parsing) we search, for each of the factors, the verbs in factor’s lists (both for the positive and negative lists);

For each of the identified verb, the subject is retrieved, also taking into account passive forms where the subject is the agent;

A check for negative forms or adverbs (e.g., “incorrectly”) is performed in the identified sentence to cope with the inversion of meaning (i.e., positive verbs become negative if a negative form or adverb is present);

The application of Algorithm 1 on these sections of CEANITA reports produced a rich output (i.e., multiple TOKAI factors were found in most of the papers). To have a more comprehensive look at the results, factors’ subjects can be grouped into flight elements (i.e., aircraft, pilot, etc.) and ground elements (i.e., controller, sector, etc.); it is then possible to estimate for each CEANITA report how the five factors are distributed between the subjects, both in terms of positive and negative occurrences.

**Figure 6.**
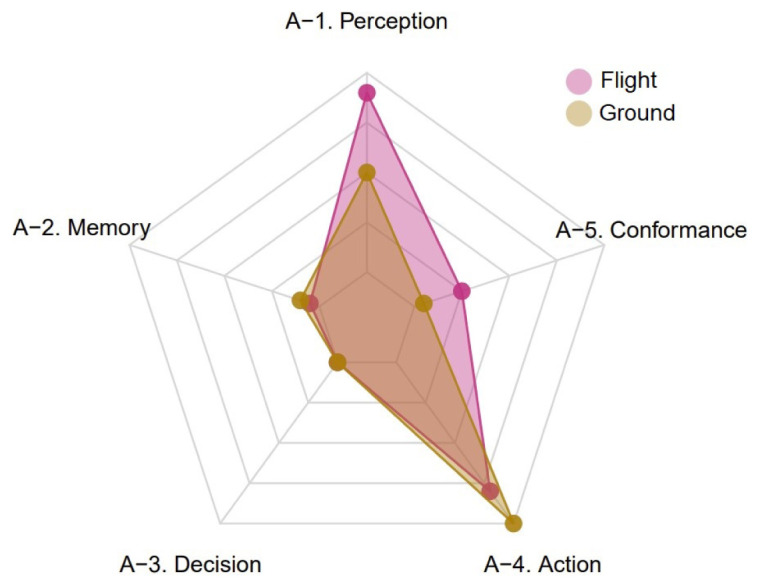
Global distribution of negative occurrences of each TOKAI-taxonomy factor by group of subjects (CEANITA reports).

Figure 6 shows the global distribution (i.e., the sum over the different reports) of negative occurrences of each TOKAI-taxonomy factor by group of subjects. Figure 6 suggests that the main omissions — namely, the actions reported in negative form in the text — for the flight subjects are classified as factor A-4 (i.e., problems with action) and A-5 (i.e., problems with conformance with rules), while for the ground subjects they are again mostly classified as factor A-4 and, to a lesser extent, as factor A-1 (i.e., problems with perception). Interestingly, further analysing the data, it is possible to discover that almost all the problems with factor A-4 are relative to (lack of) conveyance of information, for both flight and ground elements.

To assess the reliability of the proposed syntactic-analysis-based algorithm ( Algorithm 1) on CEANITA reports, an indirect validation was performed: indeed, it could not be validated in the standard way since there is no ground truth i.e., the correct classification in terms of TOKAI taxonomy does not exist for the considered reports. Thus, a simple predictive model was developed to predict the main contribution (ATCo or pilots) in a LoS, based on the extracted number of positive and negative occurrences of each taxonomy factor (i.e., the output of Algorithm 1). The idea behind this validation is that a good performance of this predictive model would indicate that the extracted information is reasonably accurate, since TOKAI taxonomy factors should well describe the ATCo’s and pilots’ contribution to the event. Specifically, for each LoS, the goal was to predict:

- the pilots’ contribution, i.e., classified as direct or not;
- the ATCos’ contribution, i.e., classified as direct or not;

based on:

- the number of positive and negative occurrences of each taxonomy factor (the outputs of Algorithm 1);
- the differences in prevalence between flight and ground subjects for each taxonomy factor;
- the airspace class (in fact, similar behaviours of ATCo and pilots can lead to different contribution assessments in different airspace classes, due to different regulations).

Note that ATCo and pilots can be both directly responsible to the incident.

Table 6 and Table 7 report the confusion matrices of the developed predictive models. In particular, a SVM with Gaussian kernel (see Section 5.4) trained with the 89 CEANITA reports was used, performing accurate model selection (the kernel and the complexity hyperparameters were searched in {10 ^−4.0^, 10 ^−3.5^, · · · , 10 ^3.0^} according to what described in Section 5.4).

**Table 6.** Confusion matrices (%) on the dummy predictive problem of estimating pilots’ direct contribution based on outputs of Algorithm 1) via SVM to validate Algorithm 1 on CEANITA reports.

		Pred.	
		No	Yes
**Truth**	**No**	51.6±0.1	12.4±0.1
	**Yes**	4.5±0.3	31.5±0.3

**Table 7.** Confusion matrices (%) on the dummy predictive problem of estimating ATCo’s direct contribution based on outputs of Algorithm 1) via SVM to validate Algorithm 1 on CEANITA reports.

		Pred.	
		No	Yes
**Truth**	**No**	25.8 *±*0.2	3.4 *±*0.2
	**Yes**	11.2 *±*0.2	59.6 *±*0.2

Confusion matrices in Table 6 and Table 7 appear reasonably balanced, especially considering that the classes are highly unbalanced. The global accuracy of the prediction is *≈*83% for pilots contribution and *≈*85% for ATCo contribution. Therefore, it can be stated that:

- the proposed approach is able to automatically link each CEANITA report to the TOKAI taxonomy factors exploiting syntactic analysis;
- the indirect validation performed through a dummy prediction problem showed promising performance supporting the quality of the proposed approach;
- as a side result of this indirect validation, the extracted link between CEANITA reports and TOKAI taxonomy appears to be a good proxy of the contribution assessment.

***6.2.2 Outcomes on UKAB reports***. Algorithm 1 has been further applied to the conclusive sections of some UKAB reports. Indeed, as the structure of UKAB reports has evolved during the years, only a subsample of them (in particular, the reports written in 2017 and a small part of the 2018 ones — the complete list is available in the output dataset ^36^) contains this free-text summary of the contributory factors.

An example of the analysed text from report 2017002 reads:

“The radar controllers did not issue timely Traffic Information. The Tac Right controller’s workload was such that he was distracted and did not sufficiently monitor the F15s. The F15 crews were not aware that AARA8 was active.”

**Figure 7.**
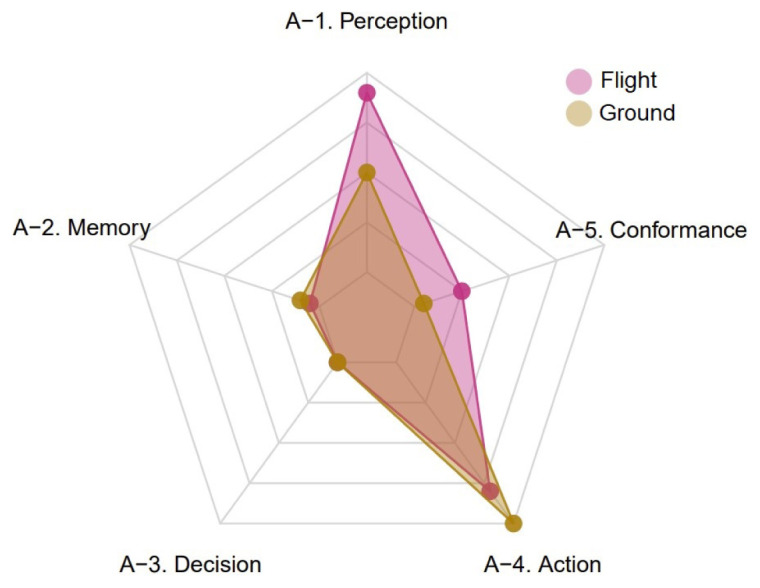
Global distribution of negative occurrences of each TOKAI-taxonomy factor by group of subjects (UKAB reports).

Despite the fact that the considered text in UKAB reports is quite different from the one in CEANITA reports, the output of Algorithm 1 is again of great interest. In particular, Figure 7 shows the global distribution of negative occurrences of each TOKAI-taxonomy factor in the selected UKAB reports by group of subjects. Figure 7 suggests that, similarly to the CEANITA use case, the most frequent factor is factor A-4, for both pilots and ATCos, while factors A-2 and A-3 are the least mentioned and factor A-5 is mostly associated with the flight subjects. Nevertheless, there is a huge difference in the prevalence of factor A-1: indeed, in UKAB reports problems with perception seem to be reported much more often, in particular for flight subjects. The comparison between Figure 6 and Figure 7 reveals another interesting detail: the shape of the yellow polygon — corresponding to the ground subjects — is almost identical in both the graphs, while the pink one differs essentially for the peak in A-1. This seems in line with what emerged in the data description ( Section 4), as the two samples of reports are very different in terms of contribution assessment: in the CEANITA corpus, the majority of the LoS events is associated to ATCo’s contribution, while, in the UKAB sample, the safety barriers suggest pilots’ actions are assessed as contributory in the vast majority of the cases and, in particular, they are mostly classified as Situational Awareness and See & Avoid problems, which may indeed be related with A-1 factor in the TOKAI taxonomy.

Analogously to the CEANITA use case, to assess the reliability of the proposed Syntactic-Analysis-based algorithm ( Algorithm 1) on UKAB reports a simple predictive model was developed. Since UKAB reports do not contain the same neat indication of ATCos’ or pilots’ contribution, two similar variables considered:

- the presence of flight safety barriers, roughly corresponding to pilots’ contribution;
- the presence of ground safety barriers, roughly corresponding to ATCos’ contribution;

according to their definition in Table 1. Similarly to the CEANITA scenario, the predictors were based on the Algorithm 1 output and on the airspace class.

Table 8 and Table 9 report the confusion matrices of the predictive models trained on the 127 UKAB reports (developed analogously to the CEANITA use case).

**Table 8.** Confusion matrices (%) on the dummy predictive problem of estimating pilots’ direct contribution based on outputs of Algorithm 1) via SVM to validate Algorithm 1 on UKAB reports.

		Pred.	
		No	Yes
**Truth**	**No**	9.1 *±*0.5	9.1 *±*0.5
	**Yes**	10.6 *±*0.5	71.2 *±*0.5

**Table 9.** Confusion matrices (%) on the dummy predictive problem of estimating ATCo’s direct contribution based on outputs of Algorithm 1) via SVM to validate Algorithm 1 on UKAB reports.

		Pred.	
		No	Yes
**Truth**	**No**	15.2±0.3	12.1±0.3
	**Yes**	12.1±0.3	60.6±0.3

Also in this case, despite the classes being highly unbalanced, the confusion matrices appear quite balanced. The global accuracy of the prediction is *≈*80% for pilots’ contribution and *≈*76% for ATCos’ contribution.

When looking at the accuracy of CEANITA and UKAB validation models, it is fundamental not to consider these numbers completely comparable, i.e., the lower accuracy of the UKAB model is not necessarily associated with a lower accuracy in the outcome of Algorithm 1 on the UKAB sample. Indeed, by reading some of the reports, it is evident that, while the CEANITA conclusive text is strictly associated with the final contribution assessment, the text considered in the UKAB reports does not focus exactly on the same aspects evaluated in the Safety Barriers assessment. Therefore, the fact that the TOKAI factors extracted from the UKAB reports are less predictive than those ones extracted from the CEANITA reports in terms of pilots’ and ATCos’ contribution might be due to the different settings of the two dummy predictive problems exploited for validation purposes.

## 7 Conclusions

The objective of this work was to facilitate the extraction of meaningful and actionable information from LoS reports and, in particular, to identify recurrent behaviours and precursors. Therefore, the authors proposed an approach based on (i) an EDA and (ii) an automatic classification of extracted knowledge considering a state-of-the-art safety taxonomy (the TOKAI one). The approach was tested on the LoS events reported in the CEANITA and UKAB public databases. For EDA purposes, unsupervised NLP techniques were applied to identify latent topics. In addition, this exploration was complemented with a clustering analysis, which facilitated the identification and grouping of similar incidents. Results demonstrated the capacity of these techniques to effectively identify meaningful topics and group together incidents, finding eight different clusters, which were assessed as valid by domain experts.

For the automatic extraction of the safety factors and their classification according to the TOKAI taxonomy, the authors leveraged syntactic analysis. This is a pioneering work in the field, and the results showed an understanding of the potential that these methods bring to safety analysis through a resilience engineering perspective. Indeed, the classification of actions according to the TOKAI taxonomy (TOKAI factors are neither negatively nor positively oriented) enables the reframing of human behaviour not as a sequence of errors that lead to an undesired outcome (i.e., only pointing out where people went wrong), but as emergent from the system, arising as a function of complex interactions. The results of this classification were validated by demonstrating the strong connection between the factors identified and the main contributor to the incident.

In the future, these techniques could be extended to other taxonomies or tailored to identify factors which should be included in the safety taxonomies, together with hidden sources of resilient performance (e.g., when not fulfilling a procedure resulted actually opportune ^37^), based on their presence on the reports, and could help facilitating the analysis pointed out in 38.

## Data availability

### Underlying data

The reports considered in this study are collected in public databases.

The considered CEANITA reports are those classified as AIRPROX, ranging from 003_18 to 071_19. The reports are available at: https://www.seguridadaerea.gob.es/es/ambitos/gestion-de-la-seguridad-operacional/ceanita#Informes%20Definitivos. The considered UKAB reports those from 2017001 to 2019335. These are available at: https://www.airproxboard.org.uk/Reports-and-analysis/Monthly-summaries/Monthly-Airprox-reviews/.

Zenodo: irene-buselli/ORE2021_14040: ORE2021_14040 v1.0 ^36^.

Data are available under the terms of the Creative Commons Zero "No rights reserved" data waiver (CC0 1.0 Public domain dedication).
