# Supplementary material for: Natural language processing for aviation safety: extracting knowledge from publicly-available loss of separation reports
Source: Open Res Eur. 2022 Feb 18;1:110. Originally published 2021 Sep 23. [Version 2] doi: 10.12688/openreseurope.14040.2 (PMC10445863; doi:10.12688/openreseurope.14040.2)
Supplement: Peer review history

# Peer review: Reviewer response for version 2

**DOI:** 10.21956/openreseurope.15560.r28603

**Published:** 2022-03-15

**Authors:** Arie Adriaensen

**Affiliations:** 1 Centre for Industrial Management/Traffic and Infrastructure, KU Leuven, Leuven, Belgium

I thank the authors for their answers to the review and for the revision of the manuscript. I am positive about the resulting additions and clarifications. Limitations of the study are now better clarified. At the same time the methodologic considerations have better been explained by additional specifications about the choice of parameters. This leads to a better defence of the final choices in the methodology.

 I am looking forward to the application of the proposed approach to more reporting data. As the authors propose, there can also be benefit in identifying forthcoming taxonomy extensions or yet-to-be-identified factors for future safety taxonomies.

Is the study design appropriate and does the work have academic merit?

Partly

Is the work clearly and accurately presented and does it cite the current literature?

Yes

If applicable, is the statistical analysis and its interpretation appropriate?

Partly

Are all the source data underlying the results available to ensure full reproducibility?

Yes

Are the conclusions drawn adequately supported by the results?

Partly

Are sufficient details of methods and analysis provided to allow replication by others?

Yes

Reviewer Expertise:

Socio-technical modelling; Human-Robot interaction; Resilience Engineering; Human Factors; Aviation Safety

I confirm that I have read this submission and believe that I have an appropriate level of expertise to confirm that it is of an acceptable scientific standard.

# Response

**Published:** 2022-03-21

**Authors:** Luca Oneto

**Affiliations:** ZenaByte, Italy

Dear Arie Adriaensen, thank you very much for appreciating our effort toward the improvement of our work. We are already trying to find a way to get more data and test our proposal.

# Peer review: Reviewer response for version 2

**DOI:** 10.21956/openreseurope.15560.r28604

**Published:** 2022-03-14

**Authors:** Riccardo Patriarca

**Affiliations:** 1 Department of Mechanical and Aerospace Engineering, Sapienza University of Rome, Rome, Italy

I truly thank the authors for their critical reflections and reconsiderations of their manuscript in light of my comments. I appreciate your efforts, and critical appraisal of the study.

 My overall impression on the revised version is positive. I agree with the frank observations by the authors about the preliminary nature of this research, and with their comment to the revised version: "It is fair to say that the NLP work package tried not to disregard the RE philosophy and to deal with complexity as much as possible, but found it often difficult to conjugate with the reporting philosophy of the data sources."

 I do believe the steps suggested in this work may motivate an extension of the repertoire of available approaches to enhance aviation safety investigations and I do look forward to future research in this area.

Is the study design appropriate and does the work have academic merit?

Partly

Is the work clearly and accurately presented and does it cite the current literature?

Partly

If applicable, is the statistical analysis and its interpretation appropriate?

Partly

Are all the source data underlying the results available to ensure full reproducibility?

Yes

Are the conclusions drawn adequately supported by the results?

No

Are sufficient details of methods and analysis provided to allow replication by others?

Partly

Reviewer Expertise:

Aeronautical engineering, air traffic management, system safety, safety risk management, resilience engineering, socio-technical systems, accident analysis, aviation safety

I confirm that I have read this submission and believe that I have an appropriate level of expertise to confirm that it is of an acceptable scientific standard.

# Response

**Published:** 2022-03-14

**Authors:** Luca Oneto

**Affiliations:** ZenaByte, Italy

Dear Riccardo Patriarca, thank you very much for appreciating our effort toward the improvement of our work. We are already thinking about possible future evolutions that we hope can further improve and empower aviation safety investigations.

# Peer review: Reviewer response for version 1

**DOI:** 10.21956/openreseurope.15131.r27674

**Published:** 2021-10-15

**Authors:** Arie Adriaensen

**Affiliations:** 1 Centre for Industrial Management/Traffic and Infrastructure, KU Leuven, Leuven, Belgium

Dear authors and editorial team,

 First of all I would like to complement the authors on the work they have performed, which shows a proposal for a novel and systematic methodology to produce meaningful interpretations out of large quantities of available reporting data. I agree that there is a general consensus within the aviation and academic community, that ironically the success of aviation reporting from the last decades also carries the responsibility of more meaningful data processing. Hence, the research topic is relevant.

**Concerns before Indexing**

 My main concern is on the relation between scope, research data and how interrelatedness might partially jeopardize the research objectives stated by the authors.

 The FARO project stands for saFety And Resilience guidelines for aviation and the manuscript’s rationale also refers to resilience principles. The use of resilience is aligned with the fact that, as the authors state, “today’s safety events are gradually becoming of a more complex and uncommon nature than those of yesteryear”; “growing amount of flights has ultimately led to increased operational complexity”, and; “increased complexity of the ATM system should be absorbed by increased deployment of automation solutions in order to achieve a more efficient and safe traffic management”.

 I agree with the authors that in order to overcome the limitation of the taxonomic approach currently used for the processing of aviation reporting, unsupervised techniques like topic modelling and similarity clustering derived from NLP can provide a great way forward. Nevertheless, the discovery of unknown patterns or further knowledge can only be as rich as the data contained in the free text of the reports. This limitation in data collection from incident investigation is typically referred to as the What-You-Look-For-Is-What-You-Find (WYLFIWYF) – principle (Lundberg 2009; Le Coze 2013). It should be considered that what Pilots and ATCos report is also the social product of the safety causation views traditionally used in the aviation community. So far, pilots are largely trained to think about safety in terms of Swiss Cheese-based & HFACS-based causation models, which are essentially expressions of safety-barrier thinking. These can be valuable models and concepts, but do not belong to the resilience engineering paradigm.

 The pilot community concepts do not necessarily produce data that are aligned with more recently developed causation models in which safety is seen as a control problem (See Leveson, 2009) or as the emergent product of functional resonance between different actions and agents (Hollnagel, 2012) to just name a few causation views that would generate different reporting data. Likewise, the understanding from system dynamics due to increased deployment of automation and increased complexity of the ATM system might not typically be covered by reports from pilots and ATCos. This is a limitation of every reporting system where typically front-end operators are expected to report, but just mentioned here to highlight the limitation of (and the impact from) this specific data source to reach a specific research objective.

 EUROCONTROL has been at the forefront of incentivising resilience-principles for many years. It could be expected that this has influenced ATCos to report differently than the pilot community, although this outside of the scope of this study.

 The arguments above are the reason why in my opinion the authors might not have reached the full objectives expressed at the beginning of the manuscript, it is still fair to say that a partial objective is reached by having increased the learning potential from existing reporting data. I suggest he authors to adjust the objectives in line with the chosen methodology. It is essentially only the process of data interpretation, which is covered by the methodology, without scrutinizing how that data could already have been influenced by the way the reporting system is constructed. Although this limitation would be true for any interpretation of reporting data, it is important in the light of reaching an increased understanding of resilience, increased operational complexity and increased deployment of automation solutions.

 The chosen data is readily available and accessible, which is very helpful for readers and reviewers.

**METHODOLOGY**

 The authors have done a great job in complementing different data processing techniques to reach a multi-perspective analysis of a fixed set of reports.          

 The created algorithm reveals a possible limitation of specific safety causation models, which previously has been addressed in the data gathering. While it is certainly preferable to differentiate occurrences between positive and negative occurrences of each taxonomy factor as the authors have done, certain causation views would even go further and say that some occurrences are neither positive or negative, but only produce positive or negative effects depending on the surrounding elements in the system. The functional resonance principle as described by Hollnagel (2012) is such an example. This is not to say, that this principle necessarily needs to be used because it essentially dismisses a taxonomic approach, but it does highlight the possibility that certain words might also be neutral or contextual. Whereas workload intuitively is only used when workload was high, communication for example can be very descriptive and produce systemic effects that are harder to capture. Are there any limitations that appeared during the clustering techniques used, which were not captured or misinterpreted by NLP techniques?

 In the light of methodological choices, it would be beneficial if the authors could defend their choice of single words and bigrams in the clustering analysis. It is not clear if the authors considered other options like for example proximity search clustering, or other additional techniques like exclusion criteria or weighing for topological modelling.

 It is interesting to see that the words, bigrams and resulting topics from the cluster analysis reveal clear differences and similarities between the CEANITA and UKAB reports. Have the researchers hypothesised why these differences appear? Is It believed to be due to a difference in the reporting culture, or in the actual causal relations that triggered he reports? More importantly, could at any point anything be learned from the difference between the CEANITA and UKAB outputs to improve the NLP learning?

 My choice to only assign a partial pass for the statistical analysis and its interpretation is only about the fact that more options could be described or the chosen options could be more clearly defended.

**ADDITIONAL REMARK**

 It seems that the polygons for Figure 6 (CEANITA reports)  and Figure 7 (UKAB reports) are identical. Although the manuscript announced that the polygons are nearly identical for ground subjects, it also announces and essential difference (a peak in A1) for flight subjects. Could the authors verify if one figure has accidentally been used for both regions?

Is the study design appropriate and does the work have academic merit?

Partly

Is the work clearly and accurately presented and does it cite the current literature?

Yes

If applicable, is the statistical analysis and its interpretation appropriate?

Partly

Are all the source data underlying the results available to ensure full reproducibility?

Yes

Are the conclusions drawn adequately supported by the results?

Partly

Are sufficient details of methods and analysis provided to allow replication by others?

Yes

Reviewer Expertise:

Socio-technical modelling; Human-Robot interaction; Resilience Engineering; Human Factors; Aviation Safety

I confirm that I have read this submission and believe that I have an appropriate level of expertise to confirm that it is of an acceptable scientific standard, however I have significant reservations, as outlined above.

## References

[1] : FRAM, the Functional Resonance Analysis Method: Modelling Complex Socio-technical Systems. *Routledge* .2012;

[2] : What have we learned about learning from accidents? Post-disasters reflections. *Safety Science* .2013;51(1) : 10.1016/j.ssci.2012.07.007 441-453 10.1016/j.ssci.2012.07.007

[3] : A new accident model for engineering safer systems. *Safety Science* .2004;42(4) : 10.1016/S0925-7535(03)00047-X 237-270 10.1016/S0925-7535(03)00047-X

[4] : What-You-Look-For-Is-What-You-Find – The consequences of underlying accident models in eight accident investigation manuals. *Safety Science* .2009;47(10) : 10.1016/j.ssci.2009.01.004 1297-1311 10.1016/j.ssci.2009.01.004

# Response

**Published:** 2022-01-24

**Authors:** Luca Oneto

**Affiliations:** ZenaByte, Italy

**Dear Arie Adriansen, we thank you for your valuable comments that allowed us to improve the manuscript.  What follows is the pointwise response to your comments with the corresponding changes in the new version of the manuscript. (Reviewer comments in italics)**

*Dear authors and editorial team, First of all I would like to complement the authors on the work they have performed, which shows a proposal for a novel and systematic methodology to produce meaningful interpretations out of large quantities of available reporting data. I agree that there is a general consensus within the aviation and academic community, that ironically the success of aviation reporting from the last decades also carries the responsibility of more meaningful data processing. Hence, the research topic is relevant.*

**We thank the reviewer for their positive comments.** **The following is a pointwise response to your comments with the corresponding changes in the new version of the manuscript.**

*My main concern is on the relation between scope, research data and how interrelatedness might partially jeopardize the research objectives stated by the authors.*

*The FARO project stands for saFety And Resilience guidelines for aviation and the manuscript’s rationale also refers to resilience principles. The use of resilience is aligned with the fact that, as the authors state, “today’s safety events are gradually becoming of a more complex and uncommon nature than those of yesteryear”; “growing amount of flights has ultimately led to increased operational complexity”, and; “increased complexity of the ATM system should be absorbed by increased deployment of automation solutions in order to achieve a more efficient and safe traffic management”. I agree with the authors that in order to overcome the limitation of the taxonomic approach currently used for the processing of aviation reporting, unsupervised techniques like topic modelling and similarity clustering derived from NLP can provide a great way forward. Nevertheless, the discovery of unknown patterns or further knowledge can only be as rich as the data contained in the free text of the reports. This limitation in data collection from incident investigation is typically referred to as the What-You-Look-For-Is-What-You-Find (WYLFIWYF) – principle (Lundberg 2009; Le Coze 2013). It should be considered that what Pilots and ATCos report is also the social product of the safety causation views traditionally used in the aviation community. So far, pilots are largely trained to think about safety in terms of Swiss Cheese-based & HFACS-based causation models, which are essentially expressions of safety-barrier thinking. These can be valuable models and concepts, but do not belong to the resilience engineering paradigm. The pilot community concepts do not necessarily produce data that are aligned with more recently developed causation models in which safety is seen as a control problem (See Leveson, 2009) or as the emergent product of functional resonance between different actions and agents (Hollnagel, 2012) to just name a few causation views that would generate different reporting data. Likewise, the understanding from system dynamics due to increased deployment of automation and increased complexity of the ATM system might not typically be covered by reports from pilots and ATCos. This is a limitation of every reporting system where typically front-end operators are expected to report, but just mentioned here to highlight the limitation of (and the impact from) this specific data source to reach a specific research objective. EUROCONTROL has been at the forefront of incentivising resilience-principles for many years. It could be expected that this has influenced ATCos to report differently than the pilot community, although this outside of the scope of this study. The arguments above are the reason why in my opinion the authors might not have reached the full objectives expressed at the beginning of the manuscript, it is still fair to say that a partial objective is reached by having increased the learning potential from existing reporting data. I suggest the authors to adjust the objectives in line with the chosen methodology. It is essentially only the process of data interpretation, which is covered by the methodology, without scrutinizing how that data could already have been influenced by the way the reporting system is constructed. Although this limitation would be true for any interpretation of reporting data, it is important in the light of reaching an increased understanding of resilience, increased operational complexity and increased deployment of automation solutions. * 

**Author Response:** **We concur with these considerations. We acknowledge these limitations and concur with the fact that they were indeed not explicit enough on the paper.** **Action Undertaken: This subject has now been mentioned in the Introduction, discussed further when commenting in the Results (end of Section 6.1) and then more thoroughly discussed in the Discussion and Conclusions (Section 7)**.

*The chosen data is readily available and accessible, which is very helpful for readers and reviewers. The authors have done a great job in complementing different data processing techniques to reach a multi-perspective analysis of a fixed set of reports.*

**Author Response:** **We thank the reviewer for his positive comments.**

*The algorithm reveals a possible limitation of specific safety causation models, which previously has been addressed in the data gathering. While it is certainly preferable to differentiate occurrences between positive and negative occurrences of each taxonomy factor as the authors have done, certain causation views would even go further and say that some occurrences are neither positive or negative, but only produce positive or negative effects depending on the surrounding elements in the system. The functional resonance principle as described by Hollnagel (2012) is such an example. This is not to say that this principle necessarily needs to be used because it essentially dismisses a taxonomic approach, but it does highlight the possibility that certain words might also be neutral or contextual*.

**Author Response:** **Given that the matter raised by the reviewer is clear and relevant, this is just to clarify that the classification of taxonomy-factor occurrences as positive and negative has to be seen, and is intended,  in a purely “grammatical” sense: “he did not comply with procedures” is classified as negative and “he followed the instruction” as positive accordingly to the verb form. However, this is not to any extent an evaluation or judgement on the positive or negative value of the action or of its effects: for instance, not fulfilling a procedure may result in a positive outcome (as pointed out in the Conclusions) and in general any evaluation of a behaviour strictly depends on the context [1].** **This does not completely solve the problem as, of course, when interpreting a graph of this kind the natural tendency is to make the grammatical meaning coincide with the “effective” one, but it has to be borne in mind that this may indeed not be the case. Action Undertaken:** **This clarification has now been added in the paper (Section 6.2.1)**

*Whereas workload intuitively is only used when workload was high, communication for example can be very descriptive and produce systemic effects that are harder to capture. Are there any limitations that appeared during the clustering techniques used, which were not captured or misinterpreted by NLP techniques?*

**Author Response:** **This limitation is acknowledged and the syntactic analysis part (to link reported actions and TOKAI factors) was intended exactly to try and overcome this limitation: while “communication” is neutral, identifying TOKAI factors like “the pilot did not communicate the position” or “the ATCo correctly provided traffic information” gives a higher level of information.** **Of course, this raises the previously mentioned problem about how to consider the impact of negative/positive sentence in a proper way, as already pointed out.** **Action Undertaken: A short paragraph about this has been added at the beginning of Section 6.2.**

*In the light of methodological choices, it would be beneficial if the authors could defend their choice of single words and bigrams in the clustering analysis. It is not clear if the authors considered other options like for example proximity search clustering, or other additional techniques like exclusion criteria or weighing for topological modelling.*

**Author Response:** **A number of preliminary analyses were performed to understand the relevance and usefulness of different n-grams to describe the reports’ content (both per se and in the topic-modelling framework), and in both CEANITA and UKAB use cases the role of n-grams with n>2 resulted substantially negligible per se and source of additional noise.** **As the reviewer suggests, we considered other (often more complex) alternatives to the proposed one, but the increasing complexity was not balanced by actual differences in the results, so we tried to keep the approach as simple as possible in order to easily allow for inspection and modification. Action Undertaken:** **This clarification has now been added in Section 5.1.**

*It is interesting to see that the words, bigrams and resulting topics from the cluster analysis reveal clear differences and similarities between the CEANITA and UKAB reports. Have the researchers hypothesised why these differences appear? Is It believed to be due to a difference in the reporting culture, or in the actual causal relations that triggered he reports? More importantly, could at any point anything be learned from the difference between the CEANITA and UKAB outputs to improve the NLP learning?*

**Author Response: We agree with the fact that both similarities and differences between CEANITA and UKAB reports are interesting to observe. Unfortunately, it is not easy to identify when the differences are due to the reporting culture (and to the different nature of the philosophy and processes of the two different investigation actors) or to the actual dynamic of the incident, nevertheless we agree it is appropriate to add some considerations about it. Action Undertaken:** **Some considerations about that have been added at the end of Section 6.1.**

*My choice to only assign a partial pass for the statistical analysis and its interpretation is only about the fact that more options could be described or the chosen options could be more clearly defended.*

**Author Response: We  recognise and acknowledge  the reviewer’s point.** **Action Undertaken: Some considerations about the reasons behind the choices of the methodologies (and more some additional specifications about the choice of parameters) were added in Section 5.1, 5.2, 5.4.**

*It seems that the polygons for Figure 6 (CEANITA reports)  and Figure 7 (UKAB reports) are identical. Although the manuscript announced that the polygons are nearly identical for ground subjects, it also announces and essential difference (a peak in A1) for flight subjects. Could the authors verify if one figure has accidentally been used for both regions?*

**Author Response: We thank the reviewer for noticing it, there was indeed a problem (Figure 7 had been accidentally duplicated and used for both). Action Undertaken: Figure 6 has been substituted with the corrected figure.**

# Peer review: Reviewer response for version 1

**DOI:** 10.21956/openreseurope.15131.r27673

**Published:** 2021-10-11

**Authors:** Riccardo Patriarca

**Affiliations:** 1 Department of Mechanical and Aerospace Engineering, Sapienza University of Rome, Rome, Italy

Dear authors, Dear editorial team,

 I thank you for giving me the opportunity to review this manuscript. I believe the presented topic is timely and the methodologies suggested by the authors are definitely at the pace with modern machine learning developments. Nonetheless, I have noticed several shortcomings from an epistemological point of view, besides observations and comments linked to the methodological development itself.

 I am documenting here my main concerns to support a critical revision of the proposed manuscript.

**MAJOR CONCERN**

*“authors show how their proposal is able to automatically extract meaningful and actionable information from safety reports and to classify them according to the TOKAI taxonomy. The quality of the approach is also indirectly validated by checking the connection between the identified factors and the main contributor of the incidents."*

 About this statement, I am here reflecting on the actual possibility to reach this target. More specifically, the two mentioned database for reporting (and please note that this comment would apply to any set of organizations) might have different ATM expertise, or operational competences, different training, different backgrounds, different organizational culture even. While there is a common interest in standardising the investigation reports, it is largely acknowledged in the practitioners community that we are not there yet. From the report we see, the authors have suggested they want to follow Resilience Engineering principles (cf. the principle of “equivalence of successes and failures”), but is the data source they are using actually able to support that view?

 How did the authors confirm the investigations they are using have been actually conducted in light of systemic (Resilience Engineering- driven) principles? Did they prove CEANTIA and UKABE (but again, I believe the same issue would apply to many other organizations) reports have been written following this logic? Are the reports actually capturing the complexity of reality? Since it does not seem the authors had any verification/validation in this sense, there is a risk that the analysis would just superimpose explanatory factors (coming from TOKAI) that actually are reliant on a different conceptual philosophy. It would have been different if the narrative comes from the same source where TOKAI factors come, at least to guarantee uniformity. Otherwise, this classification could even diverge into the side-effects typical of behaviourism.

 With respect to aviation, one could reflect upon the inter-consensus issues documented even in the case the exact same taxonomy is used (in this case HFACS):

 Nikki S. Olsen, Steven T. Shorrock, Evaluation of the HFACS-ADF safety classification system: Inter-coder consensus and intra-coder consistency, Accident Analysis & Prevention, Volume 42, Issue 2, 2010, Pages 437-444, ISSN 0001-4575, https://doi.org/10.1016/j.aap.2009.09.005

 These issues have been discussed in different domains, see for example:

 Jonas Wrigstad, Johan Bergström, Pelle Gustafson, One event, three investigations: The reproduction of a safety norm, Safety Science, Volume 96, 2017, Pages 75-83, ISSN 0925-7535, https://doi.org/10.1016/j.ssci.2017.03.009.

 I believe that’s a fundamental issue with the paper, which jeopardises the integrity of the results and conclusions. I believe the authors did not document properly these aspects and the related limitations.

*"Each CEANITA report concludes with a free-text description of the actions performed by air traffic controllers (ATCos) and pilots that contributed to the incident. Analogously, in a sample of UKAB reports, the final assessment of cause is listed in free text and includes the main contributory factors based on pilots’ and controllers’ actions. […] the main causes: the most frequent ones in the corpus are wrong clearance (52%), deviation from procedures (22%), wrong or no resolution (17%–15%), […] this section briefly summarises the main causes, contributory factors and the effectiveness of Safety Barriers as identified by the Board."*

 Indeed, from these statements, it seems the reports document only the actions that “contributed” to the incident. Similarly, the identification of causes seems to be misaligned with Resilience Engineering principles. This is also proved by the main causes listed in page 4, which recalls a reductionist simplified understanding not aligned with Resilience Engineering. How much the authors believe the listed causes are socially constructed and how much are they really representative of the messy reality, trade-offs, goal conflicts operators face in everyday work? Again in TOKAI investigations narratives are paired with contributing/mitigating explanatory factors in a neutralised language. It seems risky to just look at one side of the story.

 Similarly, the barrier-based approach documented in Figure 1 (ether effective or ineffective) recalls a traditional understanding and modelling of reality (bimodal, in contrast with the principles mentioned above), and it is unclear how the authors integrate it with modern safety thinking (and Resilience Engineering mentioned in the paper). The authors seem to make extensive usage of these elements (as documented in 5.2).

 I feel Table 1 is superfluous and could have been presented jointly as an extended legend of Figure 1.

*“This section shows how the methods presented in Section 5 have been applied to achieve the scope of the work (see Section 3) demonstrating the effectiveness of the proposed approach on both the sets of data described in Section 4.”*

 Based on my previous concerns, I invite the authors to reflect whether they have been actually able to demonstrate their initial objective, or the scope/claims of the paper need to be restructured/resized to cope with what has been /can actually be achieved.

 Why did the authors select only LoS? I agree they are particularly relevant events, but was it an opportunistic choice (i.e. numerosity)? Was there any additional justification?

**METHODOLOGY**

*“a simple model is developed”.*

 What do the authors mean here? It seems to be too generic.

 I appreciate the fact that authors are using and citing mainly open-source resources for NLP analyses, however I do believe there should be a higher level of granularity for the respective detailed methodological aspects.

 The technical details and values for the usage of LDA need to be justified. Did the authors perform any sensitivity analysis (number of iterations, burn in, alpha, etc.)? Even more, in 6.1.1., why only word and bigram? How did the authors systematically decide not to use any other n-gram?

 Similarly, the way the authors describe the identification of the k topic is too generic. What kind of testing of different options and evaluation has been performed?

 Again, how did the authors scale down the topics to 12 and 23 (from respectively 27 and 60)? Is this a signal of the inaccuracies of the underlying dataset (see previous concerns) which should have prevented from subsequent analyses? How did the authors validate the final number of clusters (experts involvement is mentioned in section 6.1.1, but how many experts, which backgrounds, how did the authors measure consensus)? Considering this manual refinement, to which extent, is this approach reproducible (resources to be involved, criteria for validation, consensus, etc.).

 Similar comments apply for missing details on the Clustering description (distance criterion, quantitative criteria for clusters identification, etc.) and for the SVM paragraph (which seems to be too generic and not contextualized in the specific problem/result).

 An overall revision is needed to add the required contribution, and remove duplicated statements (e.g. on the sample size, on the organizations involved etc.)

**IMPRECISE STATEMENTS**

*"TOKAI is a general taxonomy developed by EUROCONTROL."*

 This statement is not correct. TOKAI (and its operating version e-TOKAI) includes a taxonomy, but it is a tool for investigation.

 The authors discuss the increasing demand of air traffic as a critical aspect for complexity. I would argue that it is not only the growth in demand that increases complexity. It is also about the changing nature of human work, the dynamics of interactions between humans and technologies, the way those interactions propagate at micro-meso-macro level.

*"FARO is about “the impact that an increasingly complex environment has on the system safety”."*

 However, the authors miss the opportunity to define what is complexity in the context of their research and how its definition shaped the project investigation methods and results. Are the methods actually able to capture such complexity?

*"Note that ATCo and pilots can be both directly responsible to the incident."*

 Again, if the authors are grounding their work on Resilience Engineering principles, I wonder if this is the correct wording or might be misinterpreted as oriented towards blame culture.

**FINAL OBSERVATIONS**

 I do firmly believe that we would benefit from machine learning analyses in the safety management domain. I welcome the idea proposed by the authors to use modern techniques within safety investigations, but at the same time I recommend them to be cautious in language used, and the way they document what can be actually done or how these results configure within modern and well-established safety paradigms.

Is the study design appropriate and does the work have academic merit?

Partly

Is the work clearly and accurately presented and does it cite the current literature?

Partly

If applicable, is the statistical analysis and its interpretation appropriate?

Partly

Are all the source data underlying the results available to ensure full reproducibility?

Yes

Are the conclusions drawn adequately supported by the results?

No

Are sufficient details of methods and analysis provided to allow replication by others?

Partly

Reviewer Expertise:

Aeronautical engineering, air traffic management, system safety, safety risk management, resilience engineering, socio-technical systems, accident analysis, aviation safety

I confirm that I have read this submission and believe that I have an appropriate level of expertise to confirm that it is of an acceptable scientific standard, however I have significant reservations, as outlined above.

## References

[1] : Evaluation of the HFACS-ADF safety classification system: inter-coder consensus and intra-coder consistency. *Accid Anal Prev* .2010;42(2) : 10.1016/j.aap.2009.09.005 437-44 10.1016/j.aap.2009.09.005 20159064

[2] : One event, three investigations: The reproduction of a safety norm. *Safety Science* .2017;96: 10.1016/j.ssci.2017.03.009 75-83 10.1016/j.ssci.2017.03.009

# Response

**Published:** 2022-01-24

**Authors:** Luca Oneto

**Affiliations:** ZenaByte, Italy

**Dear Riccardo Patriarca, we thank you for your valuable comments that allowed us to improve the manuscript.  What follows is the pointwise response to your comments with the corresponding changes in the new version of the manuscript.** **(Reviewer comments in italics)**

*Dear authors, Dear editorial team, I thank you for giving me the opportunity to review this manuscript. I believe the presented topic is timely and the methodologies suggested by the authors are definitely at the pace with modern machine learning developments.*

**We thank you for your positive comments.**

*Nonetheless, I have noticed several shortcomings from an epistemological point of view, besides observations and comments linked to the methodological development itself. I am documenting here my main concerns to support a critical revision of the proposed manuscript.*

*“authors show how their proposal is able to automatically extract meaningful and actionable information from safety reports and to classify them according to the TOKAI taxonomy. The quality of the approach is also indirectly validated by checking the connection between the identified factors and the main contributor of the incidents." About this statement, I am here reflecting on the actual possibility to reach this target. More specifically, the two mentioned databases for reporting (and please note that this comment would apply to any set of organizations) might have different ATM expertise, or operational competences, different training, different backgrounds, different organizational culture even. While there is a common interest in standardising the investigation reports, it is largely acknowledged in the practitioners community that we are not there yet. From the report we see, the authors have suggested they want to follow Resilience Engineering principles (cf. the principle of “equivalence of successes and failures”), but is the data source they are using actually able to support that view? How did the authors confirm the investigations they are using have been actually conducted in light of systemic (Resilience Engineering- driven) principles? Did they prove CEANTIA and UKAB (but again, I believe the same issue would apply to many other organizations) reports have been written following this logic? Are the reports actually capturing the complexity of reality? Since it does not seem the authors had any verification/validation in this sense, there is a risk that the analysis would just superimpose explanatory factors (coming from TOKAI) that actually are reliant on a different conceptual philosophy. It would have been different if the narrative comes from the same source where TOKAI factors come, at least to guarantee uniformity. Otherwise, this classification could even diverge into the side-effects typical of behaviourism.*

**Author Response: A first, probably misleading issue, in the quoted statement is that we did indeed not actually classify THEM (i.e., the reports) but we did classify IT (i.e., the information in the reports).** **Even taking into account this correction, we understand and share the reviewer’s concern: since our algorithm classifies the information included in the reports, and the reports are not written in a standardised way (and especially not accordingly to the TOKAI logic), we have to bear in mind that our algorithm is in turn not able to standardise them. We can only extract and reshape the information that is already in the reports, inheriting all the reports’ limitations. Our intention is to show how it is possible to develop a tool to extract taxonomy factors from the text. Then, we should make it more clear in the paper that the extracted factors will just describe what is written in the report, nothing more than that: we don’t claim to have a magical tool able to make reports written with completely different logics immediately comparable and standardised. We believe that this tool remains potentially useful despite these limitations. Indeed, it may even become a diagnostic tool to identify reports’ narrative issues (e.g., we may discover that the fact that some factors are not mentioned in the CEANITA or UKAB reports is actually a problem and investigation should focus more on those aspect) or a tool to understand how reporting philosophy evolves in a period of time.** **We also agree that the data sources may not be suitable to fully adopt a Resilience Engineering view: neither of the two sources can be indeed proven to systematically capture complexity, even if they are quite progressive and enlightened (UKAB in particular), but they cannot be labelled as consistent with a RE perspective. This point is acknowledged and we understand the corresponding claims made in the paper may actually be misleading. What we intended is just that, given the reports as they are, the extraction, processing and visualisation of the results is intended to take into account a RE perspective (e.g., when choosing the taxonomy, the choice of the TOKAI one was made also in that light) as much as possible.** **Actions Undertaken:** **The quoted statement has been slightly modified (see the abstract).** **Statements about RE have been mitigated (see Introduction and Conclusions).** **A paragraph was added (see Section 3.2) to make both our intentions and the approach’s limitations explicit.** **A paragraph was added in the Conclusions to discuss these limitations and possible ways to manage them in the future.**

 With respect to aviation, one could reflect upon the inter-consensus issues documented even in the case the exact same taxonomy is used (in this case HFACS): Nikki S. Olsen, Steven T. Shorrock, Evaluation of the HFACS-ADF safety classification system: Inter-coder consensus and intra-coder consistency, Accident Analysis & Prevention, Volume 42, Issue 2, 2010, Pages 437-444, ISSN 0001-4575, https://doi.org/10.1016/j.aap.2009.09.005 These issues have been discussed in different domains, see for example: Jonas Wrigstad, Johan Bergström, Pelle Gustafson, One event, three investigations: The reproduction of a safety norm, Safety Science, Volume 96, 2017, Pages 75-83, ISSN 0925-7535, https://doi.org/10.1016/j.ssci.2017.03.009. I believe that’s a fundamental issue with the paper, which jeopardises the integrity of the results and conclusions. I believe the authors did not document properly these aspects and the related limitations.

**Author Response:** **We concur with this comment, and of its significance.  Thank you for introducing the references, that at least one we are familiar with.** **Actions Undertaken: Some considerations about inter-rater reliability have been added in the Conclusions when discussing limitations.**

 "Each CEANITA report concludes with a free-text description of the actions performed by air traffic controllers (ATCos) and pilots that contributed to the incident. Analogously, in a sample of UKAB reports, the final assessment of cause is listed in free text and includes the main contributory factors based on pilots’ and controllers’ actions. […] the main causes: the most frequent ones in the corpus are wrong clearance (52%), deviation from procedures (22%), wrong or no resolution (17%–15%), […] this section briefly summarises the main causes, contributory factors and the effectiveness of Safety Barriers as identified by the Board." Indeed, from these statements, it seems the reports document only the actions that “contributed” to the incident. Similarly, the identification of causes seems to be misaligned with Resilience Engineering principles. This is also proved by the main causes listed in page 4, which recalls a reductionist simplified understanding not aligned with Resilience Engineering. How much the authors believe the listed causes are socially constructed and how much are they really representative of the messy reality, trade-offs, goal conflicts operators face in everyday work? Again in TOKAI investigations narratives are paired with contributing/mitigating explanatory factors in a neutralised language. It seems risky to just look at one side of the story. Similarly, the barrier-based approach documented in Figure 1 (ether effective or ineffective) recalls a traditional understanding and modelling of reality (bimodal, in contrast with the principles mentioned above), and it is unclear how the authors integrate it with modern safety thinking (and Resilience Engineering mentioned in the paper). The authors seem to make extensive usage of these elements (as documented in 5.2).

**Author Response:** **As previously mentioned, we agree that the reports analysed are often not aligned with RE principles, implying a series of limitations to the work. However, here a clarification is necessary: both UKAB and CEANITA reports are composed of different sections (see Section 4) and in particular:** **1) The assessment of Main causes (for CEANITA) and Barriers (for UKAB) represents a particularly synthetic and simplified piece of information, which undoubtedly implies a reductionist view. This information is mainly considered in the EDA, before the application of more insightful analyses, and one of the main objectives of our work is indeed to reach a higher-level insight about the LoSs in order to better capture the complexity of reality and not just look at one side of the story.** **2) The different free-text sections, in particular the conclusions (CEANITA) and final assessment (UKAB), are instead of a slightly different nature: especially in the CEANITA case, they summarise the dynamics of the incident in a more extensive and richer way, also outlining actions not directly contributing to the incident (which was probably written in an unclear way in the paragraph the reviewer quotes). The TOKAI algorithm and all the other NLP models were applied to these free-text sections, and indeed the RE perspective is mainly referred to this part of the work (with all the already mentioned limitations, already mitigated in the new version of the paper).** **Actions Undertaken:** **Section 3.2 has been edited to better explain the content of the free-text parts.**

*I feel Table 1 is superfluous and could have been presented jointly as an extended legend of Figure 1.*

**Author Response:** **We agree.** **Actions Undertaken: Corrected (see Figure 1).**

*“This section shows how the methods presented in Section 5 have been applied to achieve the scope of the work (see Section 3) demonstrating the effectiveness of the proposed approach on both the sets of data described in Section 4.” Based on my previous concerns, I invite the authors to reflect whether they have been actually able to demonstrate their initial objective, or the scope/claims of the paper need to be restructured/resized to cope with what has been /can actually be achieved.* **Author Response:** **We agree.** **Actions Undertaken: An extensive discussion of the work’s limitations has been added to the Conclusions. Some statements about the objectives have been slightly mitigated in the Introduction.**

*Why did the authors select only LoS? I agree they are particularly relevant events, but was it an opportunistic choice (i.e. numerosity)? Was there any additional justification?*

**Author Response:** **The FARO project selected SMIs as a large part of the consortium have specific experience on this: on one side, ENAIRE and EUROCONTROL advised on operational matters, on the other side, two of the universities involved have conducted extensive research into the dynamics and mathematical formulations of trajectory prediction and probabilistic conflict modelling.  It was a natural choice given this experience and the composition of the consortium.**

**“** *a simple model is developed”. What do the authors mean here? It seems to be too generic.*

**Author Response:** **While we agree that that sentence was a bit too generic (now it has been corrected to “a simple Machine Learning model”), further details about that model are already extensively included in the following sections (“In particular, a SVM with Gaussian kernel trained with the 89 CEANITA reports was used, performing accurate model selection - the kernel and the complexity hyperparameters were searched in {10-4, 10-3.5 ,…, 103}…”) and the methodological foundations of SVM are described in Section 5. Actions Undertaken:** **“a simple model” has been changed to “a simple Machine Learning model”.**

*I appreciate the fact that authors are using and citing mainly open-source resources for NLP analyses, however I do believe there should be a higher level of granularity for the respective detailed methodological aspects. The technical details and values for the usage of LDA need to be justified. Did the authors perform any sensitivity analysis (number of iterations, burn in, alpha, etc.)? Even more, in 6.1.1., why only word and bigram? How did the authors systematically decide not to use any other n-gram?*

**Author Response: The Number of iterations and burn in were set by assessing convergence through the likelihood graphs produced by the models (i.e., numbers large enough to not see any more changes in the graph).** **Initial alpha and beta have been tuned together with k (even if default values turned out to be appropriate for both initial alpha and beta).** **A number of preliminary analyses were performed to understand the relevance and usefulness of different n-grams to describe the reports’ content  (both per se and in the topic-modelling framework), and in both CEANITA and UKAB use cases the role of n-grams with n>2 resulted substantially negligible per se and source of additional noise.** **Actions Undertaken: All these details have now been included in Section 5.1.**

*Similarly, the way the authors describe the identification of the k topic is too generic. What kind of testing of different options and evaluation has been performed? Again, how did the authors scale down the topics to 12 and 23 (from respectively 27 and 60)? Is this a signal of the inaccuracies of the underlying dataset (see previous concerns) which should have prevented from subsequent analyses? How did the authors validate the final number of clusters (experts involvement is mentioned in section 6.1.1, but how many experts, which backgrounds, how did the authors measure consensus)? Considering this manual refinement, to which extent, is this approach reproducible (resources to be involved, criteria for validation, consensus, etc.).*

**Author Response:** **The number k of topics was tuned accordingly to coherence and R2 metrics, searching the parameter in {10,11,…,99,100}.** **The final number of topics was manually chosen according to experts’ opinion (mainly from CRIDA, Lund University, and ENAIRE): some topics were discarded as too similar between each other, some topics because they made sense on a lexical point of view (e.g., commonly used idioms or standard sentence formulations) but did not convey any meaningful information, others because they did not make much sense or were not very coherent. To our knowledge, especially w.r.t. the R implementation we used, it is quite common to reduce the number of topics to identify the most interesting ones, and we do not consider it a signal of datasets’ inaccuracy. Since the reviewer mentions section 6.1.1 we assume he is referring to topics instead of clusters.** **When mentioning experts’ opinions, we refer mainly to those from CRIDA, Lund University, and ENAIRE, in particular involving researchers in the aviation safety and resilience fields, navigation managers and also a former ATCo. Each expert made a separate personal selection of the topics before discussing together the final one, but interestingly the single selections were not particularly different between each other, and there was a general agreement on the final selection.** **We can assume that the approach, even if not exactly reproducible due to the variability of human evaluation, could be replicated without massive differences.** **Actions Undertaken:** **All these details have now been included in Section 5.1.**

*Similar comments apply for missing details on the Clustering description (distance criterion, quantitative criteria for clusters identification, etc.) and for the SVM paragraph (which seems to be too generic and not contextualized in the specific problem/result).*

**Author Response: As reported in Section 5.2, we applied Ward’s minimum variance criterion and the criteria for cluster selection were the dendrogram, the scree plot (with distance between clusters as metrics). These two methods reduced the number of clusters to a couple of options, which were manually evaluated by the aforementioned domain experts.** **The SVM paragraph was more generic as we decided to better specify the details of the models in the following sections when describing experimental results. This was due to the fact that we found it necessary to provide some context and explain which model was applied in each different case. Details about that model were extensively included in Section 6.2.1 (“In particular, a SVM with Gaussian kernel trained with the 89 CEANITA reports was used, performing accurate model selection - the kernel and the complexity hyperparameters were searched in {10-4, 10-3.5 ,…, 103}…”). However, thanks to the reviewer’s comment, we reflected on the opportunity to move these details in Section 5 to improve the quality and the clarity of the paper.** **Actions Undertaken:** **Details about SVM have been included in Section 5.**

*An overall revision is needed to add the required contribution, and remove duplicated statements (e.g. on the sample size, on the organizations involved etc.)*

**Author Response:** **We agree (only note that the repetition of sample size was sometimes due to avoid confusion since for UKAB reports the sample sizes used for the EDA and the TOKAI algorithm is not exactly the same).** **Actions Undertaken: An overall revision has been performed.**

 IMPRECISE STATEMENTS "TOKAI is a general taxonomy developed by EUROCONTROL." This statement is not correct. TOKAI (and its operating version e-TOKAI) includes a taxonomy, but it is a tool for investigation.

**Author Response: We thank the reviewer for noticing this with this comment.** **Actions Undertaken:** **The statement has been corrected.**

*The authors discuss the increasing demand of air traffic as a critical aspect for complexity. I would argue that it is not only the growth in demand that increases complexity. It is also about the changing nature of human work, the dynamics of interactions between humans and technologies, the way those interactions propagate at micro-meso-macro level.*

**Author Response:** **Yes, we concur.  This position is also central in other FARO work packages.** **Actions Undertaken:** **Introduction has been modified to include these considerations.**

*FARO is about “the impact that an increasingly complex environment has on the system safety”." However, the authors miss the opportunity to define what is complexity in the context of their research and how its definition shaped the project investigation methods and results. Are the methods actually able to capture such complexity?*

**Author Response: In principle we agree.  A clarification about FARO is however necessary: FARO is an exploratory research project adopting a methodology that embraces orthodox safety in one work package (WP4) and a thorough study of resilient performance in a second work package (WP5). These two different perspectives of a social-technical system are contributor a third work package (WP6) which takes these two different perspectives and synthesises these to explore a view of ATM system performance. This paper reports only another – preliminary – work package (WP3) that was principally the one exploring the use of NLP on reports with the objective of capitalising extant safety knowledge. In essence whilst FARO does adopt resilience engineering precepts and principles and does focus on the impact of complexity, it is not in the specific area of interest of the task that is reported in the paper. It is fair to say that the NLP work package tried not to disregard the RE philosophy and to deal with complexity as much as possible, but found it often difficult to conjugate with the reporting philosophy of the data sources.** **To sum up: FARO indeed focuses on complexity but mainly in other parts of the project, for which this piece of research was just a starting point. Actions Undertaken: A couple of statements summarising the preliminary nature of this research have been added in the Introduction.**

***“*** *Note that ATCo and pilots can be both directly responsible to the incident." Again, if the authors are grounding their work on Resilience Engineering principles, I wonder if this is the correct wording or might be misinterpreted as oriented towards blame culture*.

**Author Response:** **We agree that the statement may be misleading. This was purely a description of what is written in the reports (i.e., there is a section about pilots/ATCos responsibilities where someone is indicated as directly responsible, possibly both ATCos and pilots) which, as already discussed, contain different parts misaligned with RE principles.** **Actions Undertaken:** **“ATCo and pilots can be both directly responsible” has been changed to “ATCo and pilots can be both indicated in the reports as directly responsible”.**

***I*** *do firmly believe that we would benefit from machine learning analyses in the safety management domain. I welcome the idea proposed by the authors to use modern techniques within safety investigations, but at the same time I recommend them to be cautious in language used, and the way they document what can be actually done or how these results configure within modern and well-established safety paradigms.*

**We thank the reviewer for his comments which helped us enhance the quality of the paper by noticing limitations and inaccuracies.**
